# Genome-wide analysis of starch metabolism genes in potato (*Solanum tuberosum* L.)

**DOI:** 10.1186/s12864-016-3381-z

**Published:** 2017-01-05

**Authors:** Jessica K. Van Harsselaar, Julia Lorenz, Melanie Senning, Uwe Sonnewald, Sophia Sonnewald

**Affiliations:** Department of Biology, Division of Biochemistry, Friedrich-Alexander-University Erlangen-Nuremberg, Staudtstrasse 5, 91058 Erlangen, Germany

**Keywords:** Starch metabolism, Gene expression, Microarray analysis, *Solanum tuberosum*, Gene annotation, Co-expression analysis

## Abstract

**Background:**

Starch is the principle constituent of potato tubers and is of considerable importance for food and non-food applications. Its metabolism has been subject of extensive research over the past decades. Despite its importance, a description of the complete inventory of genes involved in starch metabolism and their genome organization in potato plants is still missing. Moreover, mechanisms regulating the expression of starch genes in leaves and tubers remain elusive with regard to differences between transitory and storage starch metabolism, respectively. This study aimed at identifying and mapping the complete set of potato starch genes, and to study their expression pattern in leaves and tubers using different sets of transcriptome data. Moreover, we wanted to uncover transcription factors co-regulated with starch accumulation in tubers in order to get insight into the regulation of starch metabolism.

**Results:**

We identified 77 genomic loci encoding enzymes involved in starch metabolism. Novel isoforms of many enzymes were found. Their analysis will help to elucidate mechanisms of starch biosynthesis and degradation. Expression analysis of starch genes led to the identification of tissue-specific isoenzymes suggesting differences in the transcriptional regulation of starch metabolism between potato leaf and tuber tissues. Selection of genes predominantly expressed in developing potato tubers and exhibiting an expression pattern indicative for a role in starch biosynthesis enabled the identification of possible transcriptional regulators of tuber starch biosynthesis by co-expression analysis.

**Conclusions:**

This study provides the annotation of the complete set of starch metabolic genes in potato plants and their genomic localizations. Novel, so far undescribed, enzyme isoforms were revealed. Comparative transcriptome analysis enabled the identification of tuber- and leaf-specific isoforms of starch genes. This finding suggests distinct regulatory mechanisms in transitory and storage starch metabolism. Putative regulatory proteins of starch biosynthesis in potato tubers have been identified by co-expression and their expression was verified by quantitative RT-PCR.

**Electronic supplementary material:**

The online version of this article (doi:10.1186/s12864-016-3381-z) contains supplementary material, which is available to authorized users.

## Background

Potato (*Solanum tuberosum*) is one of the world’s most important crop plants. Potato tubers are rich in starch and contain minerals and vitamins as well as essential amino acids. Due to its nutrient composition and the high starch content, it serves as staple food, animal feed and feedstock for many industrial purposes including bioethanol production and food thickener. Moreover, tuber starch is used in the paper and textile industry.

**Fig. 1:**
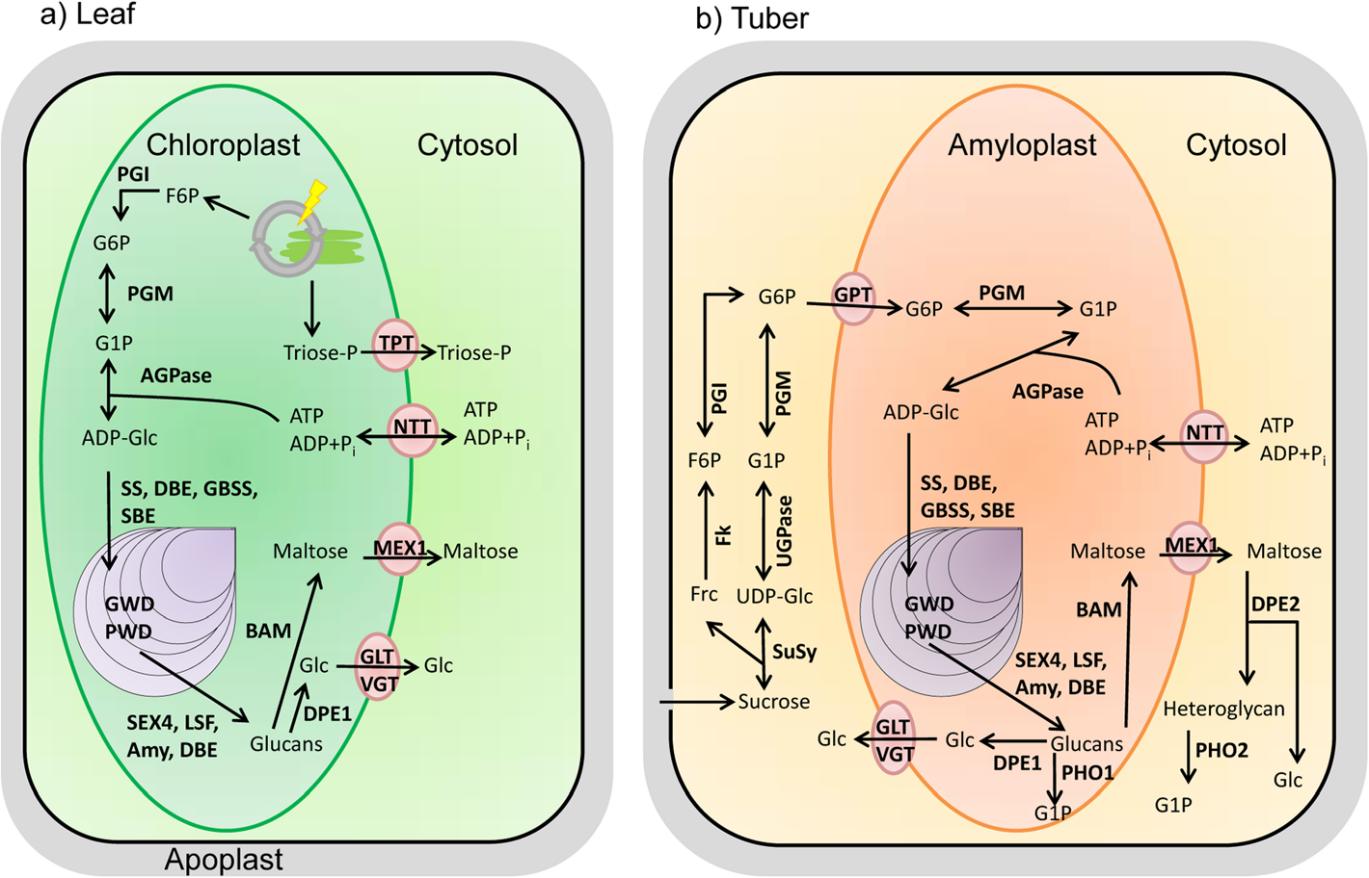
Proposed pathway of starch metabolism in leaves and tubers of potato. **a** In source leaves, photoassimilates are generated in the Calvin-Benson-Cycle. In form of F6P, these can be converted to G1P in two subsequent steps catalyzed by PGI and PGM. G1P serves as substrate for AGPase for starch biosynthesis. TPs from the Calvin-Benson-Cycle can also be transported to the cytosol via the TPT in exchange for Pi where they can be metabolized e.g. to sucrose. **b** In the tuber, sucrose is cleaved by SuSy into UDP-glucose and fructose. UDP-glucose is converted to G1P by UGPase. G1P subsequently is transferred to G6P by cytosolic PGM which can be imported into the amyloplast by GPT. In the amyloplast G6P is then reconverted into G1P by plastidial PGM and as such can serve as a substrate for starch biosynthesis. For detailed reviews on starch metabolism see [3, 4, 6] and references therein. F6P, Fructose-6-phosphate; TP, triose-phosphate; TPT, triose-phosphate/phosphate translocator; Pi, inorganic phosphate; PGI, phosphoglucoisomerase; G6P, glucose 6-phosphate; G1P, glucose 1-phosphate; PGM, phosphoglucomutase; SuSy, sucrose synthase; UGPase, UDP-glucose pyrophosphorylase; PPi, inorganic pyrophosphate; AGPase, ADP-glucose pyrophosphorylase; PPase, inorganic pyrophosphatase; SS, starch synthase; GBSS, granule-bound starch synthase; SBE, starch branching enzyme; GWD, glucan, water dikinase; PWD, phosphoglucan, water dikinase; BAM, beta-amylase; AMY, alpha-amylase; SEX4, starch excess 4; LSF, Like starch-excess Four; DPE, disproportionating enzyme; PHO, Alpha-glucan phosphorylase; GPT, glucose 6-phosphate/phosphate translocator, NTT, nucleotide translocator; GLT, glucose transporter; VGT, vacuolar glucose transporter; MEX, maltose transporter; Fk, fructokinase

Potato starch is composed of two polymers, branched amylopectin and linear amylose. Starch synthesis occurs in plastids, where both polymers form semi-crystalline, water-insoluble granules. It is generally accepted that starch is mainly synthesized from ADP-glucose through the orchestrated action of several plastid-localized enzymes [1, 2]. An overview of starch metabolism in leaves and tubers is depicted in Fig. 1 showing that there are many parallels between both tissues regarding the enzyme activities involved. Although the same overall enzymatic reactions have to be catalyzed in both, leaves and tubers, there are profound differences between both tissues. In leaves, starch is synthesized and degraded diurnally, serving as a nocturnal energy resource to maintain energy supply for biological processes. In potato tubers, starch accumulates during development and is stored over a long period of time. It maintains the energy demand of the dormant tuber and fuels the outgrowth of new shoots after dormancy is broken. In leaves, ATP needed for starch biosynthesis is generated during photosynthesis. In contrast, ATP has to be imported into the plastids in tubers. Moreover, the origin of glucosyl donors for starch biosynthesis differs between phototrophic and heterotrophic tissues. In leaf chloroplasts, the generation of ADP-glucose is directly linked to the generation of photoassimilates within the Calvin-Benson-Cycle [3]. The glucosyl donor for starch biosynthesis in sink tubers is derived from sucrose which is transported via the phloem from the photosynthetically active leaf tissues to the developing tuber. In the tuber, sucrose reaching the cytosol has to be converted to glucose 6-phosphate (G6P) which is subsequently imported into the amyloplast where it is further metabolized to ADP-Glc and starch. These differences give reasons to assume that different regulatory mechanisms operate in leaves and tubers.

Many genes coding for enzymes involved in starch metabolism are organized in gene families. Members of these families may play distinct roles in starch biosynthesis and breakdown in source and sink tissues, respectively [4]. In rice, this has been shown for isoforms of ADP-glucose pyrophosphorylase (AGPase), starch branching enzyme (SBE), starch phosphorylase (PHO), disproportionating enzyme (DPE), starch synthase (SS) and debranching enzyme (DBE) by qRT-PCR analysis of leaf and endosperm tissues [5, 6]. Regulatory mechanisms influencing activities of starch metabolic enzymes act at the post-translational level like protein-protein interactions, protein phosphorylation and redox regulation [3, 7–9]. In addition, accumulation of starch metabolic enzymes is controlled at the transcriptional level. Transcript abundance of many starch genes is regulated by the circadian clock and by sugar availability [8, 10, 11]. In *Arabidopsis* leaves, expression of the *GBSS1* gene is controlled by two clock transcription factors (TFs), namely the Myb-related CIRCADIAN CLOCK ASSOCIATED 1 (CCA1) and LATE ELONGATED HYPOCOTYL (LHY) [12], while in rice endosperm, *GBSS* was reported to be regulated by two interacting proteins belonging to the MYC and EREBP families [13]. Further evidence for transcriptional regulation of starch metabolism comes from barley, where a sugar-inducible TF, SUSIBA2, belonging to the WRKY class of TFs, bound to the promoter of the *ISA1* gene and exhibited an expression pattern similar to *ISA1* [14]. In a co-expression analysis in rice, putative regulators of starch biosynthesis were identified and subsequent functional studies showed that an APETALA2 (AP2)/EREBP-type TF negatively regulates genes involved in starch biosynthesis and is an important determinant of the starch content and structure [15]. In sweet potato, SRF1, a Dof protein, was found to have an indirect positive effect on starch biosynthesis [16]. An effect on starch gene expression was also described for FLO2 (FLOURY ENDOSPERM2) in rice seeds [17]. These examples emphasize the significance of transcriptional regulation for starch metabolism which remains largely elusive in potato.

In potato, expression of sucrose synthase (*SuSy*) and *AGPase* can be induced in response to sucrose [18, 19]. Moreover, the expression of *SuSy* and *AGPase* are high in growing tubers and decrease rapidly after detachment of potato tubers from the mother plant [9, 20]. Expression of both genes follows the diurnal rhythm in leaves and tubers [21]. These findings suggest a coordinated regulation of both transcripts [22]. Analyses of overexpression-lines and antisense-lines point to an involvement of sucrose non-fermenting-1-related protein kinase-1 (SnRK1) in the regulation of transcription of *SuSy* and *AGPase* [23, 24] but the molecular details are still unclear.

Despite the important role of potato tubers for food security, a detailed study of the genomic organization of starch metabolic genes is still missing. Based on the recently published potato genome sequence [25, 26], genes encoding enzymes involved in starch metabolism in potato were identified and annotated in this study. Thereby, so far undescribed potential starch genes were identified and a functional genomic map of the starch gene inventory of the potato was established. A comparative analysis of microarray and NGS data led to the identification of tissue-specifically expressed starch genes suggesting distinct regulatory mechanisms for leaf and tuber starch metabolism. Moreover, a co-expression analysis with tuber-specific starch genes was performed to identify transcription factors that may control starch biosynthesis in this tissue.

## Results

### Annotation of genes encoding enzymes of potato starch metabolism

In order to identify potato starch metabolism genes, a homology search using *Arabidopsis* sequences of genes previously described by Sonnewald and Kossmann [2] was conducted. Based on sequence similarity 44 out of 46 *Arabidopsis* (*Arabidopsis thaliana*) open reading frames (ORF) were assigned to homologous potato transcripts (Table 1). No homologous sequences were found for At4g24450 (GWD2), At2g21590 (APL4) and At5g17523 (similar to MEX1) in the Spud DB [27] or the NCBI databases. For all other *Arabidopsis* query sequences, a homologous sequence was found in the potato genome (Table 1).

**Table 1:** Starch metabolism genes investigated in this study

Enzyme	PGSC Gene ID	PGSC Transcript ID	iTAG Transcript ID	NCBI Reference Sequence/GenBank	Locus At
ADP-glucose pyrophosphorylase large subunit 1 (AGPL1)	PGSC0003DMG400009026	PGSC0003DMT400023304	Sotub01g024100.1.1	NM_001288466.1	At5g19220
ADP-glucose pyrophosphorylase large subunit 2 (AGPL2)	PGSC0003DMG400015952	PGSC0003DMT400041215	Sotub07g011850.1.1	NM_001318669.1	At1g27680
ADP-glucose pyrophosphorylase large subunit 3 (AGPL3)	PGSC0003DMG400000735	PGSC0003DMT400001935	Sotub01g047210.1.1	X61187.1	At4g39210
ADP-glucose pyrophosphorylase small subunit 1.1 (AGPS1.1)	PGSC0003DMG400031084	PGSC0003DMT400079823	Sotub07g023520.1.1	NM_001288195.1	At5g48300
ADP-glucose pyrophosphorylase small subunit 1.2 (AGPS1.2)	PGSC0003DMG400046891	PGSC0003DMT400097320	Sotub12g006530.1.1		At5g48300
ADP-glucose pyrophosphorylase small subunit 2 (AGPS2)	PGSC0003DMG400025218	PGSC0003DMT400064936	Sotub08g010520.1.1		At1g05610
Alpha-amylase 1.1 (AMY1.1)	PGSC0003DMG400007974	PGSC0003DMT400020591	Sotub04g031900.1.1	M81682.1	At4g25000
Alpha-amylase 1.2 (AMY1.2)	PGSC0003DMG400020603	PGSC0003DMT400053110	Sotub03g021150.1.1	A21347.1	At4g25000
Alpha-amylase 2 (AMY23)	PGSC0003DMG400009891	PGSC0003DMT400025601	Sotub04g035480.1.1	M79328.1	At1g76130
Alpha-amylase 3 (AMY3)	PGSC0003DMG401017626	PGSC0003DMT400045435	Sotub05g011310.1.1		At1g69830
Alpha-amylase 3-like (AMY3-like)			Sotub02g012780.1.1		At1g69830
Alpha-glucan phosphorylase 1a (PHO1a)	PGSC0003DMG400007782 PGSC0003DMG400003495 PGSC0003DMG400002479	PGSC0003DMT400020094 PGSC0003DMT400008970 PGSC0003DMT400006337		D00520.1	At3g29320
Alpha-glucan phosphorylase 1b (PHO1b)	PGSC0003DMG400028382	PGSC0003DMT400072963	Sotub05g005530.1.1	NM_001288199.1	At3g29320
Alpha-glucan phosphorylase 2a (PHO2a)	chr00:18163346..18176781			M69038.1	At3g46970
Alpha-glucan phosphorylase 2b (PHO2b)	PGSC0003DMG400031765	PGSC0003DMT400081273	Sotub02g020370.1.1		At3g46970
ATP-ADP antiporter 1 (NTT1)	PGSC0003DMG400005612	PGSC0003DMT400014304	Sotub03g033540.1.1		At1g80300
ATP-ADP antiporter 2 (NTT2)	PGSC0003DMG400028641	PGSC0003DMT400073724	Sotub12g021790.1.1	NM_001287865.1	At1g15500
Beta-amylase 1 (BAM1)	PGSC0003DMG400001549	PGSC0003DMT400003933	Sotub09g026990.1.1		At3g23920
Beta-amylase 2 (BAM2)	PGSC0003DMG400024145	PGSC0003DMT400062050	Sotub08g006590.1.1		At5g45300
Beta-amylase 3.1 (BAM3.1)	PGSC0003DMG400001855	PGSC0003DMT400004686	Sotub08g023010.1.1	NM_001288243.1	At4g17090
Beta-amylase 3.2 (BAM3.2)	PGSC0003DMG402020509	PGSC0003DMT400052839	Sotub08g006070.1.1		At4g17090
Beta-amylase 4 (BAM4)	PGSC0003DMG400012129	PGSC0003DMT400031627	Sotub08g027460.1.1		
Beta-amylase 6.1 (BAM6.1)	PGSC0003DMG400026199	PGSC0003DMT400067403	Sotub07g021140.1.1		At2g32290
Beta-amylase 6.2 (BAM6.2)	PGSC0003DMG400026166	PGSC0003DMT400067289	Sotub07g021110.1.1		At2g32290
Beta-amylase 6.3 (BAM6.3)	PGSC0003DMG400026198	PGSC0003DMT400067400	Sotub07g021090.1.1		At2g32290
Beta-amylase 7 (BAM7)	PGSC0003DMG400000169	PGSC0003DMT400000485	Sotub01g031940.1.1		At2g45880
Beta-amylase 9 (BAM9)	PGSC0003DMG400010664	PGSC0003DMT400027659	Sotub01g021680.1.1		At5g18670
Branching enzyme I.1 (SBE1.1)	PGSC0003DMG400022307	PGSC0003DMT400057446	Sotub07g029010.1.1		At3g20440
Branching enzyme I.2 (SBE1.2)			Sotub07g025820.1.1		At3g20440
Branching enzyme II (SBE2)			Sotub09g011090.1.1	NM_001288538.1	At2g36390
Branching enzyme III (SBE3)	PGSC0003DMG400009981	PGSC0003DMT400025846	Sotub04g035850.1.1	NM_001288254.1	At5g03650
Disproportionating enzyme 1 (DPE1)	PGSC0003DMG400016589	PGSC0003DMT400042739	Sotub04g021520.1.1	NM_001287852.1	At5g64860
Disproportionating enzyme 2 (DPE2)			Sotub02g006950.1.1	NM_001288247.1	At2g40840
Glucan water dikinase (GWD)	PGSC0003DMG400007677	PGSC0003DMT400019845	Sotub05g014130.1.1	NM_001288123.1	At1g10760
Glucose transporter (GLT1)	PGSC0003DMG400026402	PGSC0003DMT400067884	Sotub02g029320.1.1	AF215853.1	At5g16150
Glucose-6-phosphate translocator 1.1 (GPT1.1)	PGSC0003DMG400001041 PGSC0003DMG400005602	PGSC0003DMT400002701 PGSC0003DMT400014284	Sotub07g025910.1.1		At5g54800
Glucose-6-phosphate translocator 1.2 (GPT1.2)			Sotub03g008220.1.1		At1g61800
Glucose-6-phosphate translocator 2.1 (GPT2.1)	PGSC0003DMG400005269	PGSC0003DMT400013500	Sotub05g021450.1.1	AF020816.1	At1g61800
Glucose-6-phosphate translocator 2.2 (GPT2.2)	PGSC0003DMG400025495	PGSC0003DMT400065527			At1g61800
Granule bound starch synthase 1 (GBSS1)	PGSC0003DMG400012111	PGSC0003DMT400031568	Sotub08g026990.1.1	NM_001287989.1	At1g32900
Inorganic pyrophosphatase (PPase)	PGSC0003DMG400003103	PGSC0003DMT400008028	Sotub01g043620.1.1		At5g09650
Inorganic pyrophosphatase-like (PPase-like)	PGSC0003DMG400026784	PGSC0003DMT400068875	Sotub10g017670.1.1		At5g09650
Isoamylase 1.1 (ISA1.1)	PGSC0003DMG400020699	PGSC0003DMT400053345		NM_001288008.1	At2g39930
Isoamylase 1.2 (ISA 1.2)	PGSC0003DMG400030253	PGSC0003DMT400077770	Sotub10g015570.1.1	NM_001288008.1	At2g39930
Isoamylase 2 (ISA2)	PGSC0003DMG400000954	PGSC0003DMT400002502	Sotub09g015190.1.1	NM_001287875.1	At1g03310
Isoamylase 3 (ISA3)	PGSC0003DMG402007274 PGSC0003DMG401007274	PGSC0003DMT400018766 PGSC0003DMT400018765	Sotub06g007640.1.1	NM_001288291.1	At4g09020
Limit dextrinase (LDE)			Sotub11g012510.1.1 Sotub11g012520.1.1 Sotub11g012530.1.1 Sotub11g012540.1.1		At5g04360
Maltose excess 1 (MEX1)	PGSC0003DMG400024812	PGSC0003DMT400063824	Sotub04g024480.1.1		At5g17520
Phosphoglucan phosphatase (like SEX four 1, LSF1)	PGSC0003DMG400030092	PGSC0003DMT400077364	Sotub12g017200.1.1		At3g01510
Phosphoglucan phosphatase (like SEX four 2, LSF2)	PGSC0003DMG400029073	PGSC0003DMT400074765	Sotub06g009920.1.1		At3g10940
Phosphoglucan phosphatase (SEX4)	PGSC0003DMG400015246	PGSC0003DMT400039423	Sotub03g023920.1.1	NM_001318586.1	At3g52180
Phosphoglucan phosphatase (SEX4-like)	PGSC0003DMG400027327	PGSC0003DMT400070294	Sotub11g010680.1.1	NM_001318586.1	At3g52180
Phosphoglucan water dikinase (PWD)	PGSC0003DMG400016613	PGSC0003DMT400042818	Sotub09g030460.1.1	NM_001287941.1	At5g26570
Phosphoglucoisomerase (PGI)	PGSC0003DMG400012910	PGSC0003DMT400033620	Sotub04g029550.1.1	NM_001247654.3	At4g24620
Phosphoglucoisomerase-like 1 (PGI-like1)	PGSC0003DMG400015341	PGSC0003DMT400039665	Sotub12g005010.1.1	NM_001288294.1	At5g42740
Phosphoglucoisomerase-like 2 (PGI-like2)	PGSC0003DMG400030128	PGSC0003DMT400077470			
Phosphoglucomutase 1 (PGM1)			Sotub03g007170.1.1	NM_001288352.1	At5g51820
Phosphoglucomutase 2.1 (PGM2.1)			Sotub07g017160.1.1	NM_001288404.1	At1g23190
Phosphoglucomutase 2.2 (PGM2.2)	chr04:35711900..35685400				At1g23190
Putative Phosphoglucomutase (pPGM)			Sotub05g017780.1.1		At1g70820
Starch Synthase I (SS1)	PGSC0003DMG402018552	PGSC0003DMT400047731	Sotub03g013130.1.1	NM_001288145.1	At5g24300
Starch Synthase II (SS2)	PGSC0003DMG400001328	PGSC0003DMT400003356	Sotub02g034860.1.1	NM_001288048.1	At3g01180
Starch Synthase III (SS3)	PGSC0003DMG400016481	PGSC0003DMT400042496	Sotub02g023740.1.1	X94400.1	At1g11720
Starch Synthase IV (SS4)	PGSC0003DMG400008322	PGSC0003DMT400021444	Sotub02g017380.1.1		At4g18240
Starch Synthase V (SS5)	PGSC0003DMG400030619	PGSC0003DMT400078688	Sotub02g030260.1.1	NM_001288111.1	At5g65685
Starch Synthase VI (SS6)	PGSC0003DMG402013540	PGSC0003DMT400035218	Sotub07g015820.1.1	NM_001247458.1	
Sucrose Synthase 1 (SuSy1)	PGSC0003DMG400013547	PGSC0003DMT400035264	Sotub07g016120.1.1		At5g20830
Sucrose Synthase 2 (SuSy2)	PGSC0003DMG400013546	PGSC0003DMT400035262	Sotub07g016110.1.1	NM_001287982.1	At5g49190
Sucrose Synthase 3 (SuSy3)	PGSC0003DMG400006672	PGSC0003DMT400017087		NM_001288308.1	At4g02280
Sucrose Synthase 4 (SuSy4)	PGSC0003DMG400002895	PGSC0003DMT400007506	Sotub12g008670.1.1	M18745.1	At3g43190
Sucrose Synthase 6 (SuSy6)	PGSC0003DMG400031046	PGSC0003DMT400079728	Sotub03g023000.1.1		At1g73370
Sucrose Synthase 7 (SuSy7)	PGSC0003DMG400016730	PGSC0003DMT400043117	Sotub02g024410.1.1		At5g37180
Triose-phosphate/phosphate translocator (TPT)	PGSC0003DMG400022832	PGSC0003DMT400058772	Sotub10g009470.1.1	NM_001287896.1	At5g46110
Triose-phosphate/phosphate translocator-like (TPT-like)			Sotub01g020040.1.1		At5g46110
UDP-glucose pyrophosphorylase 1 (UGPase1)			Sotub05g026990.1.1		At3g03250
UDP-glucose pyrophosphorylase 2 (UGPase2)	PGSC0003DMG401013333	PGSC0003DMT400034699	Sotub11g007290.1.1	NM_001288019.1	At5g17310
Vacuolar Glucose Transporter 3-like (VGT3-like)	PGSC0003DMG401010374	PGSC0003DMT400026885	Sotub03g022010.1.1		At5g59250

For the identification of isoforms of starch metabolic enzymes, a keyword search in the Spud DB database was undertaken using the enzyme names as queries. Additionally, manually corrected potato transcript sequences resulting from the homology and keyword searches were re-BLASTed against the potato genome and the sequences of second best hit were analyzed to identify putative isoforms. This led to the discovery of two genes which had not been annotated, namely *PGM2.2* and *PHO2a. PGM2.2* could be assigned to chromosome 4 while *PHO2a* was located on an unanchored scaffold.

Eventually, predicted transcript sequences of all identified genes were compared to published mRNA sequences available on the NCBI data base via a BLAST search. Sequence alignments were conducted to check for completeness of the ORFs and the predicted protein sequences. The exon-intron structure of the genes was manually re-annotated and/or corrected, if required. Correct assignment of potato transcripts compared to the corresponding *Arabidopsis* orthologs was verified by protein sequence comparison. Phylogenetic trees were constructed using the translated ORF sequences of all putative members of a gene family. Phylogenetic trees of selected gene families are depicted in Additional file 1. If ambiguities were encountered, a motif search was conducted using the online tool MEME [28]. The presence and order of motifs was compared between sequences assuming a high degree of similarity between members of the same gene family [29]. If this was the case the identified gene was considered as an isoform.

Application of the above mentioned strategies resulted in the identification of 77 loci coding for enzymes of starch metabolism in potato (Table 1). In comparison to *Arabidopsis*, additional putative isoforms of AGPS1, PHO1 and PHO2, TPT, BAM3, BAM6, SBE1, GPT1 and GPT2, PPase, ISA1, SEX4, PGM2, PGI, AMY1 and AMY3 were found. The deduced transcripts of BAM6.2, BAM6.3, SBE1.2 and ISA1.2 were highly identical to their respective paralogs but did not seem to comprise full-length transcripts. This might be either a result of an incorrect genome assembly or incomplete gene duplication events.

**Fig. 2:**
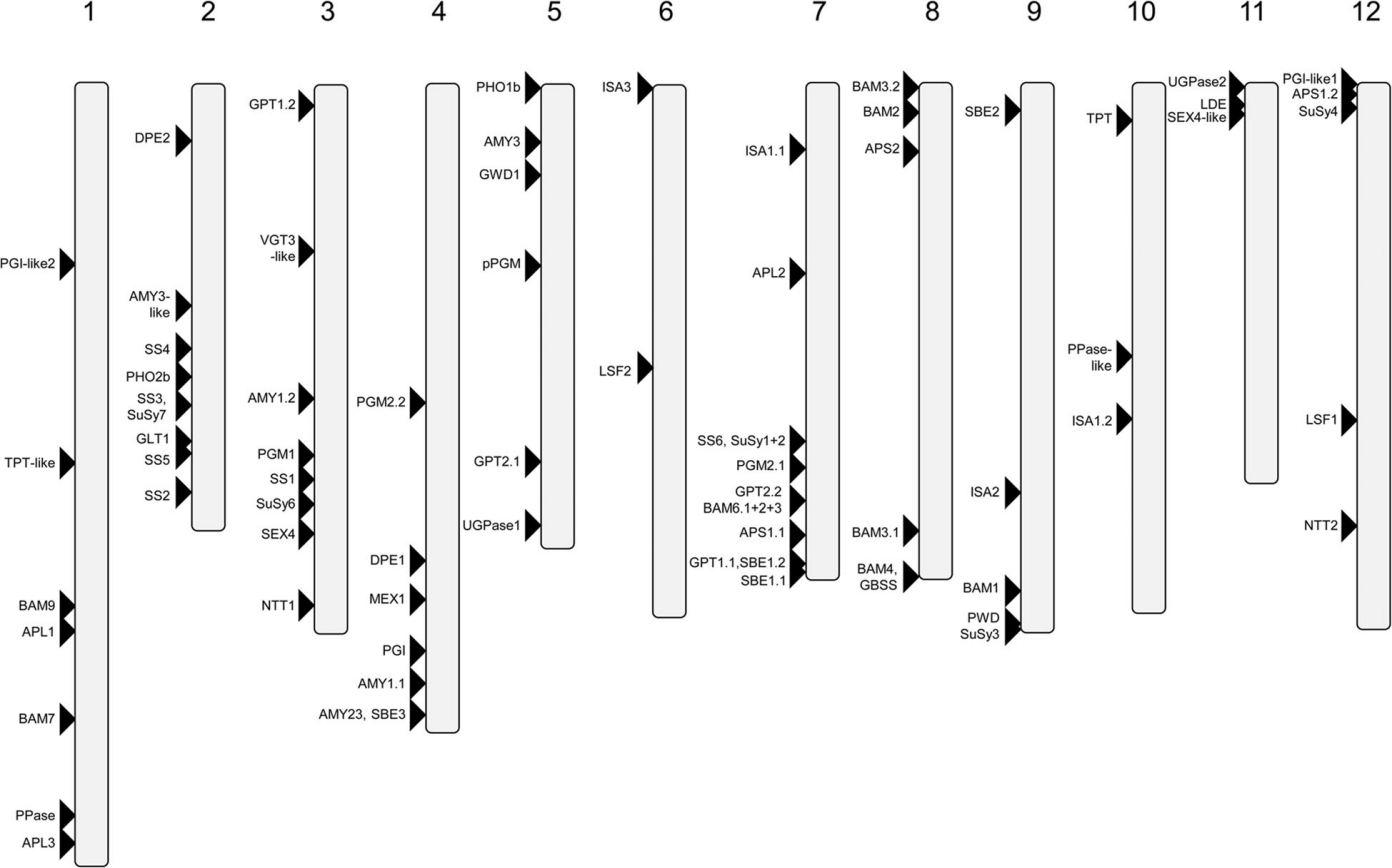
Ideogram of physical positions of starch metabolism enzymes in the potato genome. The relative map positions of 75 genes encoding starch metabolism genes are shown on the individual pseudomolecules depicting the chromosomes 1–12

Chromosomal positions of putative starch genes were retrieved from the Spud DB genome browser v4.03 [30] and visualized using the location-based display tool on the Ensembl plants website [31, 32]. Manual editing allowed the visualization of genes as an ideogram (Fig. 2). For two genes, *PHO1a* and *PHO2a*, no physical position could be defined since their genes are located on unanchored scaffolds, but orthologous sequences from tomato are located on chromosomes 3 and 9, respectively. This is in accordance with results from quantitative trait loci (QTL) analyses in potato that mapped two glucan-phosphorylases to those chromosomes [33, 34].

Figure 2 shows that genes coding for starch metabolism enzymes are distributed over all twelve potato chromosomes. There is a concentration of *SS* (*SS2*, *SS3*, *SS4*, *SS5*) on chromosome 2 and many genes encoding BAMs are located on chromosome 8. SuSy and ISA encoding genes are distributed across different chromosomes. An interesting finding was the discovery of two *PGM2* isoforms *PGM2.1* and *PGM2.2* which are located on chromosome 7 and 4, respectively. The sequence identity between both transcripts is 99.5%, but the corresponding genes differ significantly in their non-coding regions showing only 59% sequence similarity. However, the structure of both genes appears to be conserved. The *PGM2.2* isoform has not been predicted by the PGSC or iTAG and was identified by BLASTing the transcript sequence Sotub07g017160.1.1 against the scaffold sequences. Investigating the tomato genome available on the Sol Genomics website (https://solgenomics.net [35]) for *PGM2* genes revealed that only one locus is present which is localized on chromosome 4. Therefore, it is conceivable that the *PGM2.1* gene on chromosome 7 is the result of a recent gene duplication event, however this needs to be further investigated by bioinformatics analysis.

### Identification of suitable microarray identifiers to investigate gene expression

Two oligonucleotide-based microarray platforms (Agilent Technologies) are available for global gene expression analysis in potato. The POCI array was designed in 4x44k format based on a collection of expressed sequence tags (EST) [36] while the 8x60k microarray is based on predicted transcript sequences of the DM potato genome by the PGSC [37]. In this study, experimental data of both microarray designs were used. The prerequisite for the comparative expression analysis was the identification of suitable microarray oligonucleotides (identifiers) matching the transcript of interest, particularly in case of the POCI platform. Therefore, prior to expression analysis, oligonucleotide binding accuracy to the target genes was assessed. To this end, transcript and genomic sequences of starch genes were BLASTed against the POCI database [38] and resulting EST sequences were aligned to the genomic sequence to allow for assessment of their corresponding oligonucleotide binding capacities to the transcript. Oligonucleotides matching the reference sequence with 85% or more identity were considered for the analysis of expression profiles. Due to the lack of matching EST-sequences or to binding of the corresponding oligonucleotides within predicted introns, no suitable oligonucleotides were found for *AMY3-like*, *AGPS1.2*, *AGPS2*, *TPT*, *TPT-like*, *GPT1.2*, all *BAM6* isoforms, *BAM7*, *BAM9*, *PGI-like2* and *pPGM* in the POCI platform.

Since oligonucleotide sequences of the 8x60k microarray were deduced from predicted transcript sequences of the DM genome, they perfectly match the corresponding transcript available at the Spud DB website. In these cases the position of the oligo within the gene was assessed to rule out that the binding site is within a putative intron. Oligonucleotide specificity was investigated by multiple sequence alignments. The high sequence similarity between the transcripts of some isoenzymes prevented the assignment of specific oligonucleotides discriminating the isoforms of *ISA1*, *SEX4-like*, *SBE1* and *BAM6.2* and *BAM6.3*. Additional file 2 lists all identifiers from both platforms that met our criteria and that were considered for further analyses.

### Identification of genes that are highly expressed in leaves or tubers

**Fig. 3:**
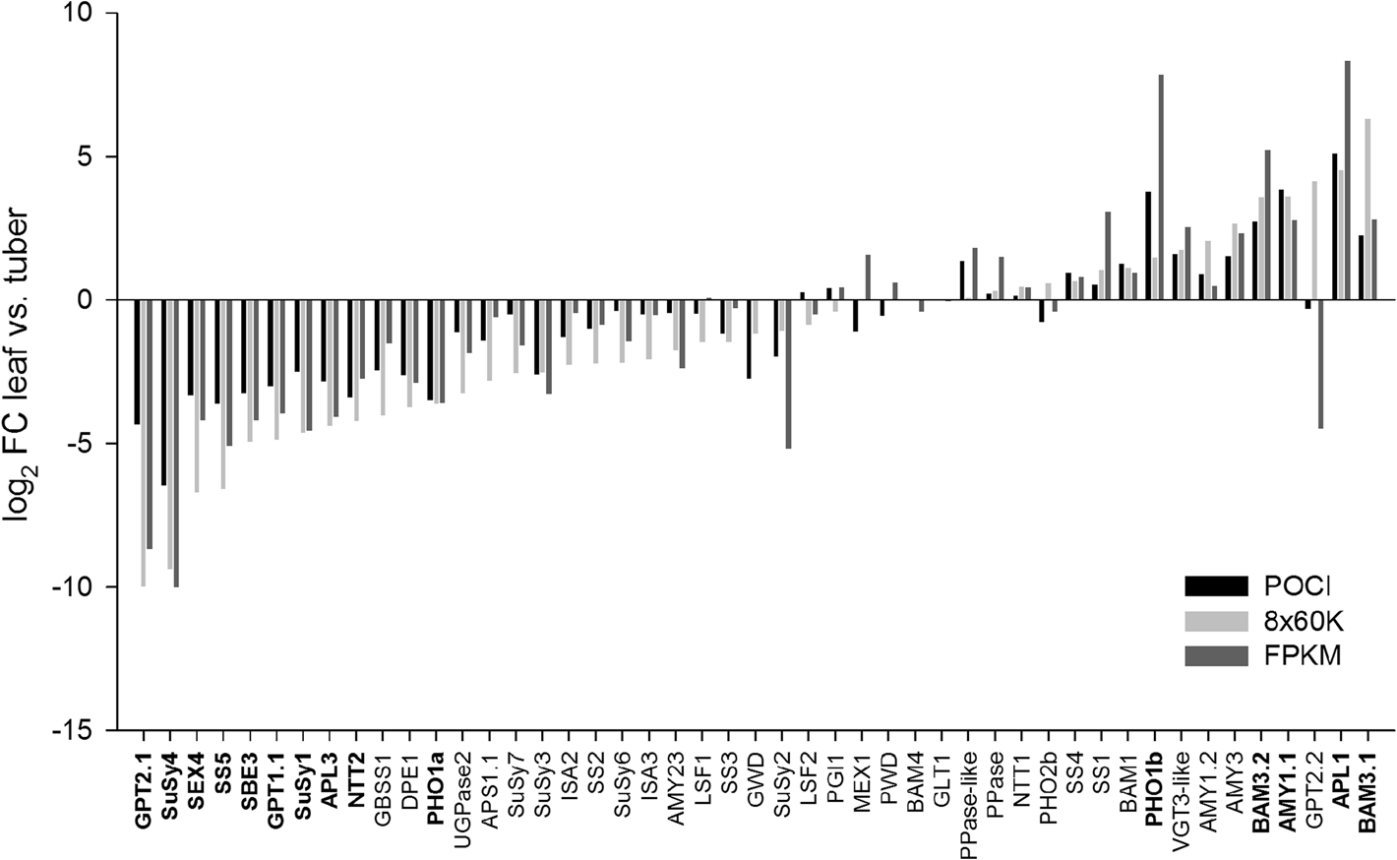
Relative expression of starch genes in leaf vs. tuber tissue. Fold-change values for individual genes between leaf and tuber samples were exported from GeneSpring or calculated from FPKM values from the PGSC database. Genes whose expression cannot be detected in either platform were excluded. Light grey bars: values from 8x60 microarray, black bars: values from 4x44k POCI array, dark grey bars: FPKM values

For the gene expression analysis, samples taken from leaf and tuber tissues were selected from different microarray experiments (Additional file 3). Raw data files of the different samples were uploaded into the GeneSpring 12.6.1. GX software and were normalized together. Direct comparisons of gene expression were made within the individual platforms first. Afterwards derived results were compared between the different platforms. To identify starch genes that are preferentially expressed in leaves or tubers, the fold-change between the mean relative expression value detected in leaf and tuber samples was calculated using the GeneSpring 12.6.1. GX software and displayed in Additional file 4. For genes, whose expression was ascertainable in both microarray platforms, the log2 fold-change was calculated and depicted in Fig. 3. We considered genes that were on average more than 10-fold overexpressed in one tissue to be tissue-specific. The comparison between the two array platforms revealed that several genes are specifically expressed in leaves or tubers, respectively (Fig. 3). Hence, a strong tuber-specific expression was detected for *GPT2.1* and *SuSy4* followed by *SEX4* and *SS5*, whereas *BAM3.1*, *APL1* and *AMY1.1* were found to be highly expressed in leaves. Fold-change differences between leaf and tuber samples were often greater in the 8x60k array than in the POCI array but the tendency was similar (Additional file 4). The only exception was *GPT2.2* whose expression was unchanged between leaf and tuber samples hybridized onto the POCI array but showed a 17.5-fold higher expression in leaves than in tubers in samples analyzed on the 8x60k array (Additional file 4).

To confirm our results, FPKM (Fragments Per Kilobase Of Exon Per Million Fragments Mapped) values of corresponding genes were downloaded from RNA-sequencing data available on the Spud DB website and leaf and tuber samples were selected. Ratios between leaf and tuber values were calculated and compared to the results from the microarray analyses. Fold-change values of the RNA-Seq data compared well to the microarray data (Fig. 3, Additional file 4). Thus, *GPT2.1* and *SuSy4* are highly tuber-specifically expressed genes. Their expression was 20- to 1000-fold higher in tubers compared to leaves. Leaf-specific expression of *AMY1.1*, *APL1* and *BAM3.1* could also be confirmed by the RNA-Seq data. They were found to be 7-fold to 320-fold higher expressed in leaves than in tubers (Additional file 4).

**Fig. 4:**
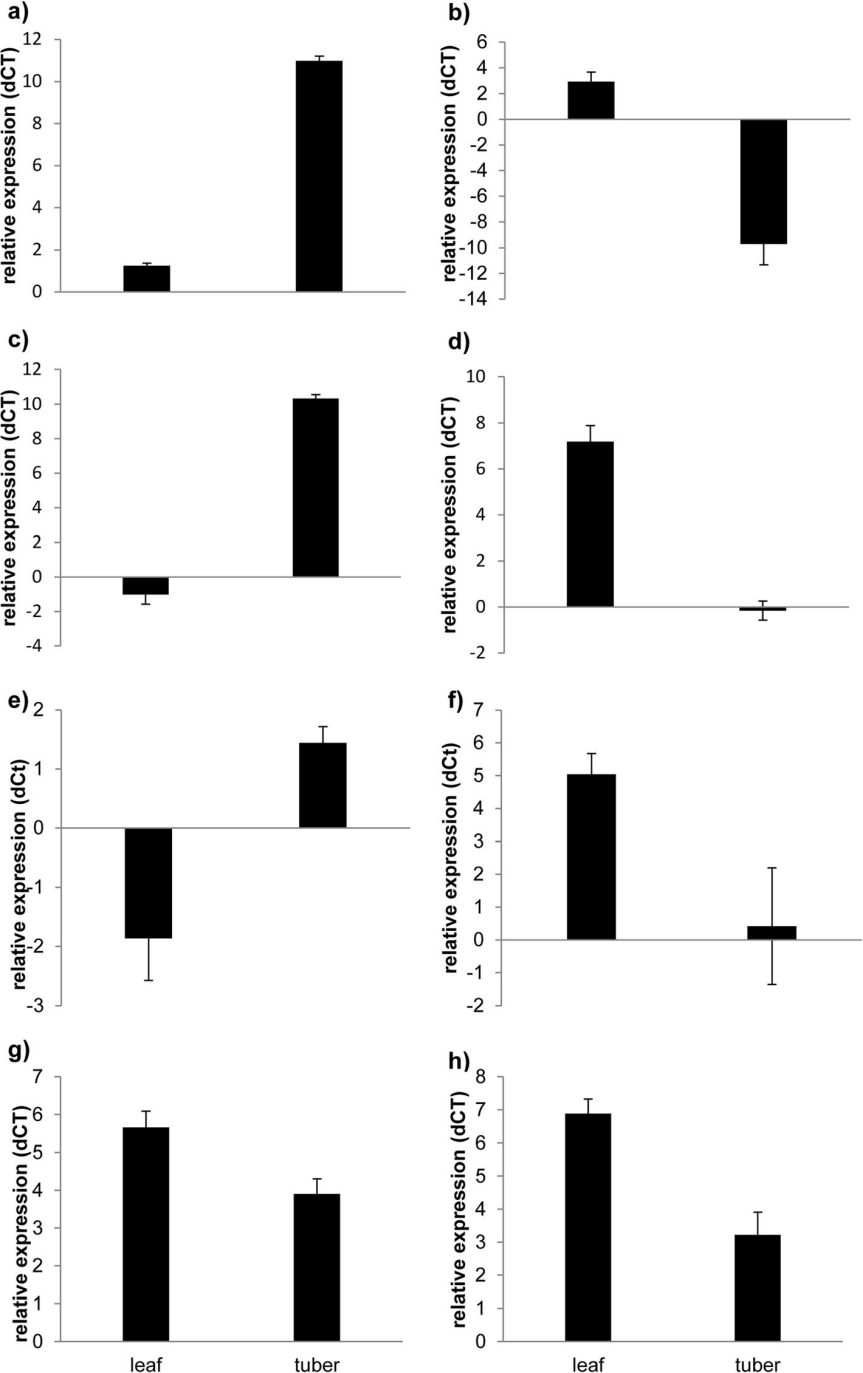
qRT-PCR analysis of selected starch metabolism genes in potato leaves and tubers. Plants were grown in a greenhouse for 11 weeks until harvest and sampling. Mean relative expression of four biological replicates normalized to EF1alpha is illustrated as dCT-value of **a** SuSy4, **b** APL1, **c** GPT2.1, **d** BAM3.1, **e** SS5, **f** AMY1.1, **g** LSF2, **h** APL2. Error bars represent standard deviation

Verification of differential expression of selected genes was carried out by quantitative real-time PCR (qRT-PCR). As shown in Fig. 4, tuber-specific expression was confirmed for *SuSy4*, *GPT2.1* and *SS5* as well as the leaf-specific expression of *AMY1.1*, *APL1* and *BAM3.1* (Fig. 4a-f). In addition, we selected two genes, *APL2* and *LSF2*, showing a similar expression in leaves and tubers in all three transcriptome platforms. Again, qRT-PCR analysis confirmed the transcriptome data (Fig. 4g, h).

### Selection of query genes for co-expression analysis

**Fig. 5:**
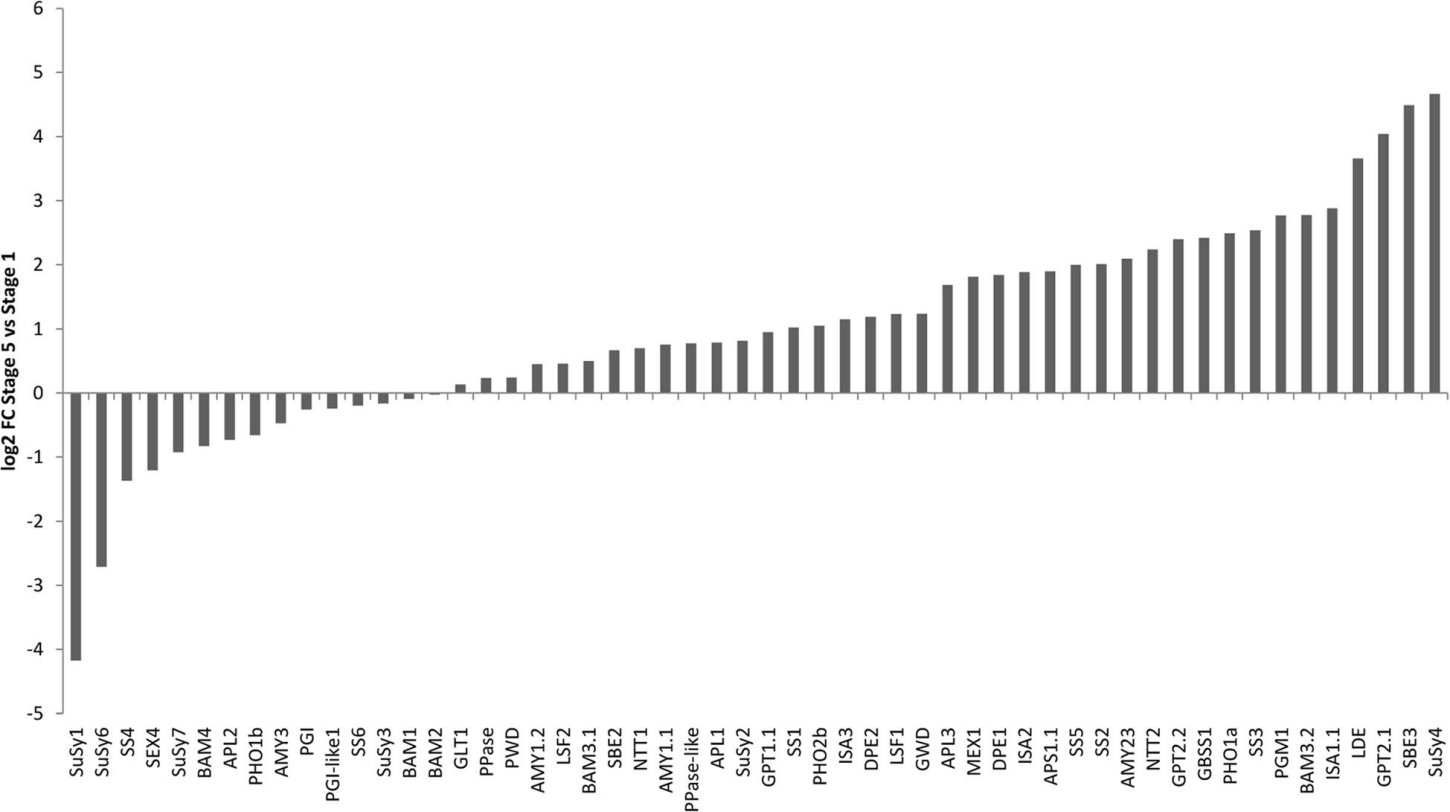
Relative changes in expression of starch genes during tuber development (stage 5 vs. stage 1). Given are log2 transformed fold-changes. Data were taken from Ferreira et al. 2010 [20]

The main goal of the co-expression analysis (see below) was to identify possible regulators of starch biosynthesis in potato tubers. Therefore, the genes used as queries for the analysis were selected by two criteria; first, they had to be specifically expressed in the tuber and second, their expression pattern had to follow starch accumulation. The first criterion was fulfilled most strongly by *GPT2.1*, *SuSy4*, *SEX4*, *SS5* and *SBE3* (Fig. 3). For the evaluation of the second criterion, increasing gene expression during tuber development was chosen. It is known that during tuberization the rate of starch biosynthesis increases significantly [39]. Therefore, genes involved in starch biosynthesis should be upregulated during this process. To identify these genes, microarray data from the tuber induction experiment described by Ferreira et al. [20] were inspected and the ratio of transcripts detected in small tubers (stage 5) vs. those measured in unswollen stolons (stage 1) were calculated and illustrated as log2 values (Fig. 5). The highest up-regulation from stage 1 to stage 5 was seen for *SuSy4*, *SBE3*, *GPT2.1* and *LDE. SEX4*, which was identified as specifically expressed in tubers, showed a pronounced down-regulation in the course of tuber development (Fig. 5). Therefore, *SuSy4*, *SBE3* and *GPT2.1* were chosen as query genes for the co-expression analysis.

### Co-regulation analysis to identify putative regulators of starch metabolism in potato tubers

**Fig. 6:**
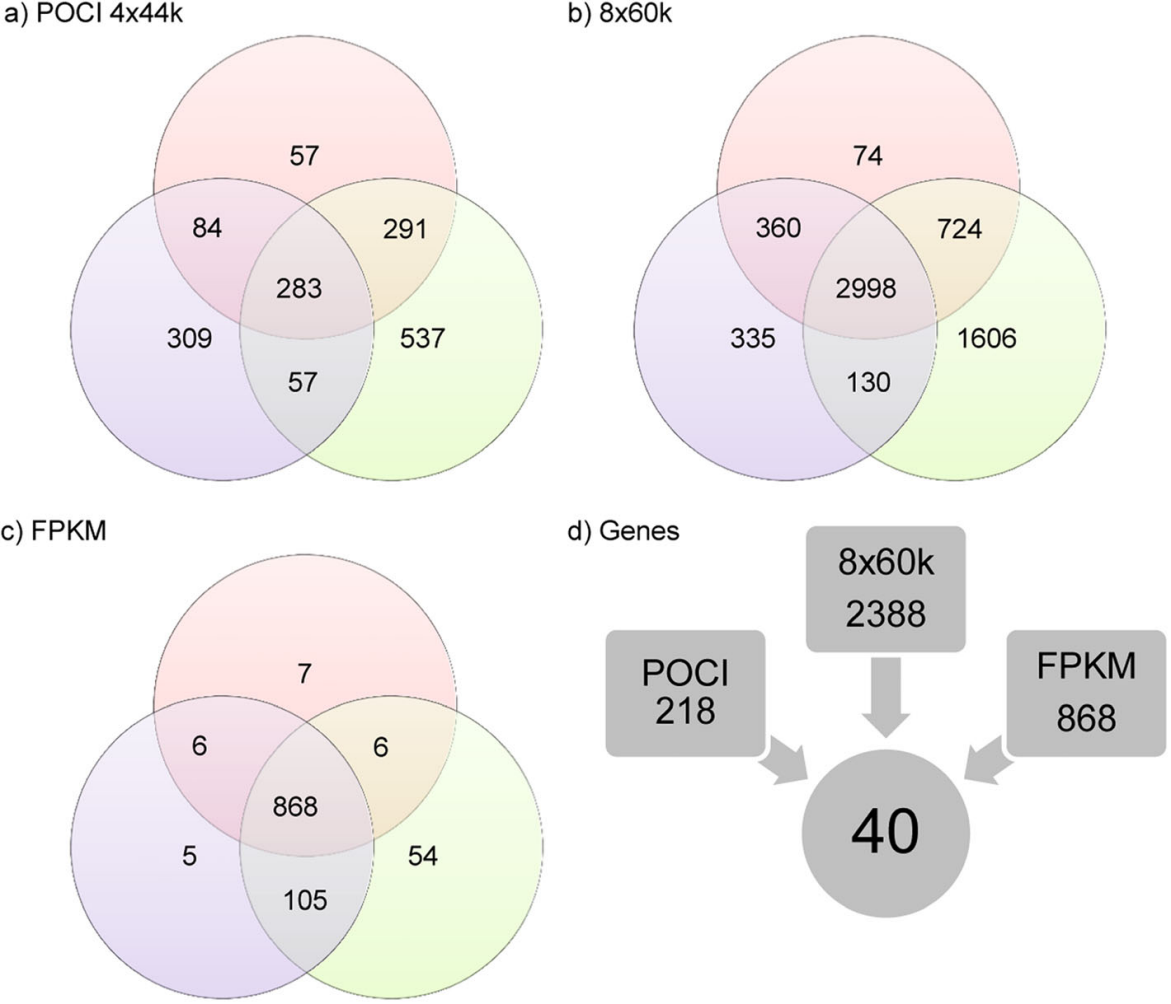
Overview of co-expression analysis. **a-c** Venn-analysis of co-expressed entities with *SuSy4* (red circles), *SBE3* (blue circles) and *GPT2.1* (green circles). Co-expression analysis was conducted using a PCC cut-off of 0.8 < =r < =1.0. **a** co-expressed entities in the POCI microarray platform, **b** co-expressed entities in the 8x60k microarray platform, **c** co-expressed entities in the RNA-sequencing data. **d** After conversion of the co-expressed entity lists to gene lists, the lists were compared and the common genes in all three lists were retrieved

To identify possible regulators of starch biosynthesis in potato tubers, all valid microarray identifiers for each of the selected genes (see Additional file 2) were used as queries in a Pearson correlation search on all detected entities in both microarray platforms including all data sets. In addition, RNA-Seq data were also analyzed. A Pearson correlation coefficient (PCC) of 0.8 was used as cut-off (Additional file 5). Within each platform, the overlap of entities co-expressed with all three query genes was determined using VENN diagrams (Fig. 6). The numbers of genes co-regulated with *GPT2.1*, *SuSy4* and *SBE3* differed greatly between platforms ranging between 283 entities in the POCI array, 868 for the RNA-Seq data set and 2998 in the 8x60k array (Fig. 6a-c). To compare the results from the different platforms, found entities were assigned to their corresponding PGSC gene identification number. This resulted in a list of 40 different genes that were consistently co-expressed with *GPT2.1*, *SuSy4* and *SBE3*. Besides the three query genes, five other starch genes, namely *APL3*, *PHO1a*, *SS5*, *NTT2* and *GPT1.1* were among the co-expressed genes (Additional file 5) and were identified to be tuber-specifically expressed (Fig. 3). Functional categorization of the 40 co-expressed genes revealed that twenty percent of the co-expressed genes encode known storage proteins like patatin and protease inhibitors [40].

To identify possible transcriptional regulators of starch biosynthesis in potato tubers, we paid special attention to putative TFs. Among those, TFs with homology to regulators of organogenesis from *Arabidopsis* like Petal Loss (PTL), Lateral Organ Boundaries (LOB), Blade On Petiole2 (BOP2) and Lateral Root Primordium protein (LRP) were found. Furthermore, a WRKY-type TF (WRKY4) and a member of the plant-specific TIFY (or ZIM) motif containing protein family TIFY5a, were co-expressed with the starch biosynthesis genes (Additional file 5).

**Fig. 7:**
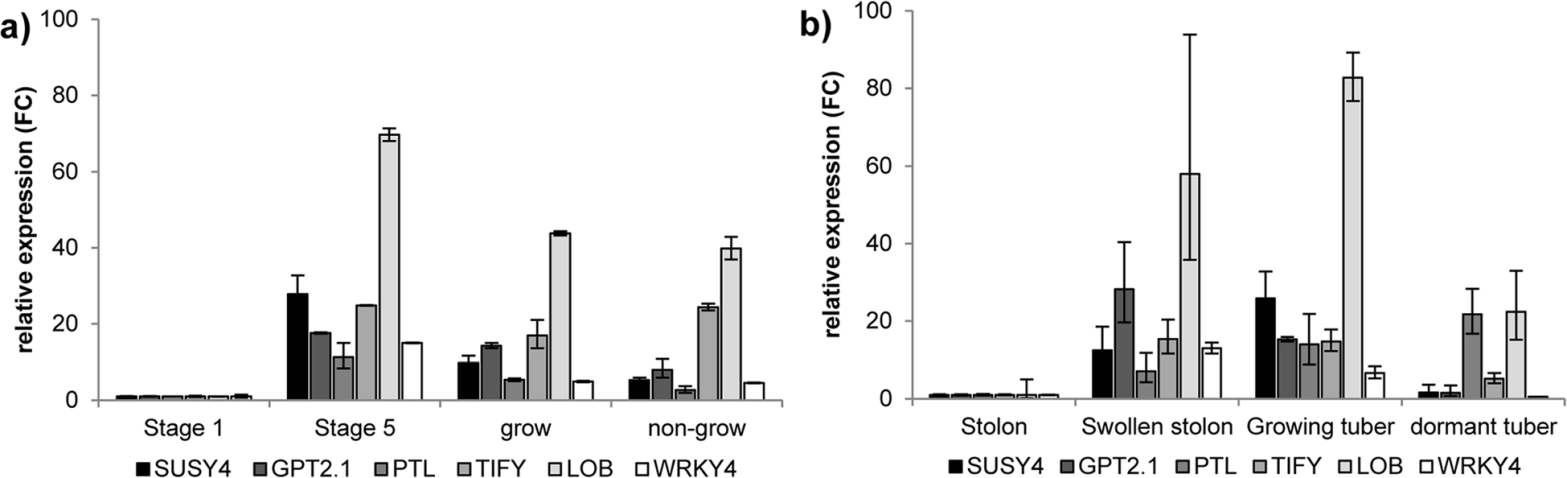
Expression profiles of tuber-specific starch genes and co-expressed transcription factors. **a** Gene expression calculated as fold-change relative to the value at stage 1 in the microarray experiments. **b** qRT-PCR analysis of the same genes in independent samples. Each value represents the mean of 3–4 biological replicates. Error bars represent standard deviation. Black bars: SuSy4, dark grey bars: GPT2.1, grey bars: PTL, medium grey bars: TIFY5a, light grey bars: LOB domain containing protein, white bars: WRKY4

To confirm the expression profiles, four putative TF (PTL, TIFY5a, LOB and WRKY4) as well as SuSy4 and GPT2.1 were selected for qRT-PCR analysis. The relative amount of the corresponding mRNA was quantified in an independent set of samples representing four different stages of tuber development, namely unswollen stolons (stage 1), swollen stolons (stage 3–5), growing tubers and dormant tubers. The results were compared to microarray data derived from similar stages of tuber development (stage 1, stage 5, growing tubers and non-growing tubers [20]). As shown in Fig. 7, the results from qRT-PCR were generally comparable to the results from microarray analysis when considering similar stages of tuber development. With respect to *SuSy4* slight differences between both techniques were observed. While its expression reached highest values in stage 5 in the microarray, a maximum transcript amount of *SuSy4* was seen in growing tubers in qRT-PCR. The expression profiles of *LOB* followed those of *SuSy4* in both setups. *WRKY4* as well as *TIFY5a* showed similar profiles in both platforms and correlated highly to the expression of *GPT2.1* (Fig. 7)*.* One exception was the expression profile of *PTL*. While its expression was lower in growing and non-growing tubers as compared to stage 5 in the microarray experiments, the mRNA level increased steadily across all developmental stages in the qRT-PCR reaching its maximum in dormant tubers (Fig. 7).

A Pearson correlation matrix was constructed evaluating the similarity of the expression profiles determined by qRT-PCR (Table 2). Most PCC values were greater than 0.6 indicating that selected genes were co-regulated in the samples analyzed by qRT-PCR. However, the PCC values were lower than in the global co-expression analysis which is most likely due to the decreased sample number. Thus, the qRT-PCR analysis corroborated that expression profiles of *TIFY5a*, *LOB* and *WRKY4* are similar to those of *SuSy4* and *GPT2.1* during tuber development. For *PTL*, the PCCs calculated from qRT-PCR were low supporting the observation that the expression pattern of this gene in the samples used for qRT-PCR deviated from the microarray.

**Table 2:** Pearson correlation coefficients between starch genes and TFs based on qRT-PCR analysis

	GPT2.1	SuSy4	PTL	TIFY5a	LOB	WRKY4
GPT2.1	1.00	0.61	−0.16	**0.90**	0.74	**1.00**
SuSy4		1.00	0.12	**0.84**	**0.96**	0.58
PTL			1.00	0.16	0.26	−0.22
TIFY5a				1.00	**0.95**	**0.88**
LOB					1.00	0.71
WRKY4						1.00

Results from the qRT-PCR analysis of starch genes and transcription factors were subjected to a Pearson correlation analysis using Microsoft Excel. Correlation coefficients with *p* ≤ 0.1 are indicated in bold letters

## Discussion

### Genome-wide analysis of starch genes in potato reveals novel isoforms

Enzymes involved in starch metabolism often belong to gene families encoding several isoenzymes. To our knowledge, this work presents the first genome-wide analysis of starch genes in potato. A comprehensive BLAST search strategy complemented by motif discovery and comparison to known sequences from *Arabidopsis* was applied aiming at the identification of all “starch gene” loci in potato. We found 77 loci coding for starch metabolism related enzymes belonging to different enzyme classes. Higher plants possess five gene classes encoding starch synthases, designated *GBSS* and *SS1-4* [4]. In rice, two forms of *GBSS* were identified and eight genes encoding the four *SS* classes [41]. In the potato genome, we confirmed that GBSS which has been reported previously to be responsible for amylose biosynthesis in the starch granule, is encoded by a single gene and is expressed higher in tubers than in leaves [42]. A second transcript (DMT400003356) annotated as *GBSS2* in the PGSC database had previously been described to possess soluble SS activity [43, 44] and was found to be the closest potato homolog to *Arabidopsis SS2* and was therefore consequently designated as SS2. Moreover, it was shown that SS2 plays only a minor role in starch biosynthesis in tubers [43] which is in accordance with our expression analysis showing only a slightly higher expression in tubers compared to leaves although being upregulated during tuber development. In total, seven starch synthases were found in the potato genome (*GBSS*, *SS1-6*) most of which have been described in earlier studies [42–48]. However, no studies have analyzed the roles of SS5 and SS6 in potato yet, but our gene expression data suggest a possible role for SS5 in potato tuber starch biosynthesis. In contrast, SS6 is expressed to similar levels in leaves and tubers (Additional file 4) and its expression was not found to change significantly during tuber development (Fig. 5). Thus, further analyses are necessary to investigate the function of these genes during starch biosynthesis in potato. A recent publication described the phylogenetic relationship of SS from different plant species, including potato, confirming the presence of a fifth class of SS [49]. In addition the authors found maize *SS5* to be highly expressed during the grain filling stage suggesting a role in starch biosynthesis [49], which is in agreement with our assumption.

In this study, enzymes were designated regarding to their annotation in *Arabidopsis*. In most cases, this was in accordance with isoform numeration of already described enzymes of potato. One exception concerns the numeration of isoforms within the SBE class where we identified four isoenzymes. Two of them share a very high sequence similarity to each other and have been denoted as *SBE1.1* and *SBE1.2* due to their homology to *Arabidopsis SBE1*. The deduced transcript sequences of these two genes, however, do not correspond to the previously published potato *SBE1* sequences [46, 50, 51]. The gene product designated *SBE1* in the aforementioned studies corresponds to *SBE3* in this study. It was described as the major SBE isoform in potato tubers and was found to play a role in starch granule formation [51, 52]. This is in accordance with the expression profile during tuber development and tissue preference discovered in this study. Until now, only variants of two isoforms, SBE3 and SBE2, have been shown to act as branching enzymes in the amyloplast [46, 52]. The role of the two potato SBE1 paralogs identified in this study remains unclear. In *Arabidopsis*, SBE1 has an effect on embryogenesis and is essential for plant growth and development [53]. A direct implication of *At*SBE1 in starch metabolism is unacquainted.

### Comparative microarray analysis revealed tissue-specific gene expression

To identify tuber- and leaf-specifically expressed starch genes different microarray data sets were analyzed. To enable the analysis, specific microarray probes had to be assigned to the different starch genes and their respective isoforms. In general, our findings were in agreement with previously published gene expression analyses and showed a high reproducibility between the two microarray platforms. Tissue-specific expression of enzyme isoforms was for example found for *PHO1a* and *PHO1b. PHO1b* appeared to be preferentially expressed in leaves, while *PHO1a* was expressed higher in tubers, which is in accordance with previous findings [54, 55]. In the case of *AGPase*, most subunits are expressed slightly higher in tubers than in leaves according to our results. However, one isoform, namely *APL1*, was clearly expressed higher in leaves than in tubers. This is in contrast to findings from La Cognata et al. [56] who described tuber-specific expression of *APL1* (designated AGP S3 in their work). The reliability of our results was confirmed by RNA-Seq data and by qRT-PCR using leaf and tuber samples. Genes showing tuber-specific expression were *SuSy4*, *SBE3*, *SS5*, *GPT2.1* and *SEX4*. In contrast to the other tuber-specific isoforms, *SEX4*-specific transcripts were not up-regulated during tuber development which is consistent with the proposed role of the enzyme in starch degradation [57]. The activity of the main SuSy isoform in tubers, SuSy4, is connected to the onset of tuberization [58–60] and correlates well with transcript and tuber starch accumulation in potato [61]. Accordingly, *SuSy4* overexpression led to an increased starch content and higher tuber yield in potato plants [62] supporting its suggested key role in starch metabolism. Similarly, *SBE3* and *GPT2.1* expression have been linked to tuber development and the accompanying accumulation of starch [63–65]. In this context, overexpression of *GPT2.1* together with *NTT* resulted in increased tuber starch content and yield [66] indicating that expression and activity of GPT2.1 are closely related. The similarity between the expression patterns of these enzymes strongly supports the assumption of a coordinated transcriptional regulation of genes within the same pathway [63]. Moreover, these examples confirm that enzymatic activity of SuSy and GPT2.1 nicely correlates with transcript accumulation and that accumulation of starch metabolic enzymes is controlled at the transcriptional level. However, in other species activity of starch metabolic enzymes was shown to be additionally regulated by post-translational mechanisms. For example, phosphorylation of SuSy isoforms was shown to influence sub-cellular localization and protein stability [67]. Activity of SBE isoforms was reported to be regulated by protein phosphorylation and redox state [68].

### Co-expression analysis reveals candidate regulators of starch biosynthesis

Co-expression analysis has previously been described to be a suitable tool for the identification of co-regulated genes [69, 70]. Assuming that proteins with regulatory functions have to be expressed at the same time or shortly before their target genes, the identification of candidate regulators should be possible by co-expression analysis. The great potential of this strategy has already been demonstrated in several studies including different plant species and tissues [15, 71–73]. One example is the identification of Rice Starch Regulator 1 (RSR1) by Fu and Xue [15] in a co-expression analysis similar to the approach used in this study. RSR1 was found to be negatively co-expressed with rice starch synthesis genes and was experimentally verified as a modulator of starch gene expression.

In this work, genes that were identified as being tuber-specifically expressed and exhibiting an expression pattern that coincides with starch biosynthesis in the potato tuber were used to search for potential transcriptional regulators, since they are so far not known. The number of genes identified to be co-expressed with *SuSy4*, *GPT2.1* and *SBE3*differed between the two microarray platforms, and was about 10 times higher in the 8x60k experiments than in those performed with the POCI platform. One reason for this might be the sample selection of the 8x60k platform which basically consists of tuber samples in similar developmental stages while most samples taken from the 4x44k format were originally designed to reflect starch biosynthesis during tuber formation. Therefore, expression profiles derived from experiments using the POCI array were expected to be more specific with respect to the identification of putative regulators of starch biosynthesis in potato tubers. Moreover, we reasoned that co-expression of a regulator with its target genes should occur in all situations. Thus, candidate selection was made after comparing the results of the co-expression analyses of the three query genes in three different platforms each with many individual samples. Eventually we identified 40 genes that are consistently co-regulated with *SuSy4*, *GPT2.1* and *SBE3*. Inspection of co-expressed genes revealed a strong over-representation of genes involved in primary carbon metabolism and development as well as genes encoding storage proteins. Tuber development and storage metabolism are known to be highly associated processes [39] which strengthens the significance of the retrieved candidates. Beside this, putative TFs co-expressed with the selected starch genes could be identified. They belong to different classes and none of them has been characterized in potato so far. Clearly, there is a strong enrichment of TFs associated with developmental processes and organogenesis like BOP2, LOB, PTL and LRP.

For *PTL*, a co-expression with *SuSy4* and *GPT2.1* in samples representing different tuber developmental stages could not be confirmed via qRT-PCR and PTL might therefore not be a good candidate for further analysis. The expression profiles obtained by qRT-PCR of the other three TF were in accordance with those of the microarray analysis (Fig. 7). Slight variations between qRT-PCR and microarray were found when comparing expression levels of *SuSy4* or *LOB* on “Stage 5” and “grow” from the microarray to “Swollen stolon” and “growing tuber” samples used for qRT-PCR. In the microarray, highest gene expression was seen on “Stage 5”, while in the qRT-PCR expression peaked in growing tubers (Fig. 7). Nevertheless, an increasing expression level was always associated with tuber formation. A possible explanation for this disagreement might be slightly different developmental stages of the samples used for the analyses. For the microarray defined stages of tuber development were sampled [20, 39], while for the qRT-PCR swollen stolons of different developmental stages were pooled. Furthermore, the growing tubers for the microarray experiment were monitored by X-ray CT analysis to determine their growth velocity, while the tubers sampled for qRT-PCR were taken from plants during their growth period, assuming that the tubers were still growing. Despite these small differences between different experiments, expression levels of *LOB*, *TIFY5a* and *WRKY4* correlate well with *SuSy4* and *GPT2.1* (Table 2). Thus they might be interesting candidates for further analyses.

In *Arabidopsis*, BOP2 and its close homolog BOP1 regulate the expression of LOB-domain containing proteins [74]. LOB expression has been found in the boundary regions between meristematic tissue and developing lateral organ primordia of the shoot apical meristem and the roots [75]. A similar spatial expression is exhibited by LRP1 of *Arabidopsis* which has been shown to be expressed in root primordia in early developmental stages [76]. In maize the localization of LRP in lateral root primordia was confirmed and it was demonstrated that LRP expression was auxin-inducible [77]. A link to auxin-signaling may also be established by the closest homolog of potato WRKY4 in *Arabidopsis*. Based on sequence similarity, the closest homolog in *Arabidopsis* is WRKY23 which has been linked to auxin-signaling in root development [78, 79]. A role of auxin in tuber initiation has been suggested [80] but a direct link to starch biosynthesis is missing. The expression patterns of these TFs suggest that there are interesting candidate genes which may directly or indirectly control starch biosynthesis and that more detailed investigation of their role is worthwhile.

## Conclusions

In this study the complete inventory of starch metabolism genes and their genomic localization was described which will facilitate future examinations of the distinct functions of isoenzymes in this pathway. Moreover, novel as far undescribed enzyme isoforms were identified whose characterization will shed more light on the mechanisms of starch biosynthesis and degradation in potato plants. Comparative microarray analysis uncovered leaf- and tuber-specific starch gene isoforms. This finding suggests distinct regulatory mechanisms in transitory and storage starch metabolism. A co-expression analysis was conducted using tuber-specific genes aiming at the identification of regulators of starch biosynthesis in potato tubers. Forty genes showed strong co-regulation in all platforms analyzed. Among the co-expressed genes were many storage metabolism genes belonging to the starch biosynthesis pathway or storage proteins as well as TFs. None of the identified TFs had been described in potato yet, but many of their homologs in *Arabidopsis* are known regulators of lateral organ development. We conclude that tuber development and tuber starch biosynthesis are highly connected pathways and consider it worthwhile to investigate the influence of the identified regulators on starch biosynthesis.

## Methods

### Identification of genes encoding starch metabolism-relevant enzymes

For the identification of genes coding for enzymes involved in starch metabolism, a list of *Arabidopsis thaliana* genes published by Sonnewald and Kossmann [2] was taken as starting point. All bioinformatics analyses, pairwise and multiple alignments, phylogenetic tree building and assembly of DNA sequences were carried out using the Geneious Pro 5.5.6 software [81]. Arabidopsis sequences were BLASTed against the potato scaffold sequences (*S. tuberosum* Group Phureja DM1-3 Version 3 DM scaffold sequences) to identify homologous sequences complemented by a keyword search on the PGSC website (http://solanaceae.plantbiology.msu.edu/index.shtml). Genomic sequences from homology searches and transcript sequences from keyword searches were compared by pairwise alignments. For verification of identified loci, predicted transcript sequences were BLASTed against the NCBI non-redundant nucleotide collection using the MEGABLAST search algorithm to find matching transcript sequences that have already been described. BLAST search was also conducted against the EST database on the NCBI website (http://blast.ncbi.nlm.nih.gov/Blast.cgi) as well as on the POCI website [38] and resulting sequences were aligned to the genomic sequences. A motif search was conducted using the MEME online tool (meme-suite.org) [28] and motifs were compared between sequences within the same gene family.

Identification of suitable microarray features was based on the alignments mentioned above. Oligonucleotide sequences were annotated to the PGSC transcript sequences or to the POCI ESTs and the matching binding site within the predicted transcript was analyzed for sequence similarity. Features represented by oligonucleotides binding in predicted introns were discarded and a threshold of 85% sequence similarity was applied for oligonucleotides to be accepted as valid for further analyses of microarray data.

### Plant material and growth conditions

Growth conditions of *Solanum tuberosum* plants from previously published experiments (no. 1–3 and 6, see Additional file 3) are described in Ferreira et al. 2010 [20] and Hancock et al. 2014 [37]. The cultivar Solara (Bioplant, Ebstorf, Germany) was used for the analysis of dormant buds and sprouts (experiments no. 4 and 5) as well as for qRT-PCR analysis. The cultivar Agria (SAKA Pflanzenzucht GmbH & Co. KG, Windeby, Germany) was used for the heat experiments (no. 7 and 8). All plantlets were propagated in tissue culture on MS-Medium [82] containing 2% (*w/v*) sucrose under conditions of 16 h light (150 μmol m^−2^ s^−1^) and 8 h dark at 21 °C. For the analysis of dormant buds, sprouts and for qRT-PCR analysis, plantlets of cv. Solara were transferred to individual 20 cm pots containing soil into the greenhouse, under conditions of 16 h light (250–300 μmol m^−2^ s^−1^) at 21 °C and 8 h dark at 18 °C and a relative humidity of 50% for three months. Samples for qRT-PCR analysis were taken on the day of harvest after 11 weeks of plant growthor at different stages during tuber development. Dormant buds were sampled from tubers stored in the dark at room temperature for one week after harvest. Tuber sprouts (sprout length ~1 mm) were collected after 12 weeks of storage. Per replicate 8–10 tubers were sampled, corresponding to 60–80 buds or sprouts, and frozen in liquid nitrogen. Plantlets of cv. Agria for experiment no. 7 were grown under the same conditions. For the heat treatment, plants grown for 6.5 weeks in the greenhouse were transferred to a phytochamber for a 7-day period under 16 h light (250–400 μmol m^−2^ s^−1^) at 29 °C and 8 h dark at 27 °C and a relative humidity of 70%. Subsequently, plants were transferred back to greenhouse conditions for 2 weeks of recovery. Leaf samples for microarray analysis of control and heat treated plants were taken after 6.5 (before heat), 7.5 (end of heat period) and 9.5 (harvest) weeks from five leaves of five individual plants per replicate. Tuber samples were taken after 9.5 weeks from tubers looking normal and tubers showing a second growth phenotype (primary and secondary tubers) from individual tubers of different plants. Agria plantlets for experiment no. 8 were transferred to 10.5 cm pots containing soil in the phytochamber, under conditions of 8 h light at 21 °C and 16 h dark at 19 °C for 30 days for accelerated tuber induction. After tuber induction, day length was changed to long day conditions for a period of 10 days. At the end of the experimental growth period leaf and tuber samples were taken and frozen in liquid nitrogen and were stored at −80 °C until further analysis.

### RNA isolation

RNA was isolated as described previously [20, 37, 83]. Total RNA was quantified and quality checked using the ND-1000 Spectrophotometer (NanoDrop Technologies).

### cDNA synthesis and qRT-PCR analysis

Two μg of total RNA were treated with DNase I (Thermo Scientific) prior to reverse transcription using oligo d(T) primers and RevertAid™ H minus first strand cDNA synthesis kit (Thermo Scientific) according to the manufacturer’s instructions. For relative quantification of starch gene derived transcripts, qRT-PCR analyses were performed using the Mx3000P qPCR system (Agilent Technologies) in combination with the Brilliant II SYBR® Green QPCR Master Mix (Agilent Technologies) with four biological replicates for each tissue and two technical replicates. EF1α was used for normalization of target gene expression. The thermal profile was as follows: 1 cycle 10 min at 95 °C for DNA polymerase activation followed by 40 cycles of 30 s at 95 °C, 30 s 60 °C and 30 s 72 °C and subsequently a melting curve. Primers were designed using the Primer-designing tool on the NCBI website [84] to have a product length ranging from 70–150 bp and a melting temperature from 59-61 °C. Sequences are given in Additional file 6.

### Microarray hybridization

Total RNA was purified using RNeasy Mini Spin Columns (QIAGEN, Valencia, CA) and integrity was verified using an Agilent 2100 BioAnalyzer (vB.02.03 BSI 307). cDNA and cRNA synthesis was performed as described in the one-color microarray-based gene expression analysis protocol provided by Agilent including the one-color RNA spike-in kit (Agilent Technologies, Santa Clara). After fragmentation, Cy3-labelled samples were loaded on the arrays and hybridized over-night (17 h/65 °C). Slides were washed as recommended in the manufacturer’s protocol and scanned on the Agilent Microarray Scanner with extended dynamic range at high resolution. Data sets were extracted with the feature extraction software (Agilent Technologies) using a standard protocol.

### Data analysis

Data files of all experiments were imported into GeneSpring 12.6.1 GX software. Experiments conducted in POCI array format included the tuber buds, tuber sprouts, leaf samples from a diurnal time course, samples taken at different stages during tuber induction and growing and non-growing tubers (Additional file 3). Samples from experiments conducted with 8x60k arrays included control leaves and tubers at 0, 8 and 12 h from Hancock et al. [37], leaf samples taken 6.5, 7.5 and 9.5 weeks after planting from control and heat treated plants as well as tuber samples at harvest, and leaf and tuber samples from experiment no. 8 (Additional file 3). All samples from one platform were normalized together applying default settings comprising log2 transformation, per chip normalization to the 75^th^ percentile and feature baseline correction to the median of all samples.

In order to identify leaf or tuber specifically expressed genes, an interpretation called “tissue” was created in the GeneSpring12.6.1 GX software grouping all samples from leaf tissue and all samples from stolon, tuber and sprout tissue together (considered as “tuber”). This was done for each microarray platform separately. Subsequently the ratio between leaf and tuber was calculated giving the fold-change difference in gene expression between the two tissues for all individual starch genes. Genes exhibiting an average absolute fold-change above 10 were regarded as being tissue-specifically expressed.

For co-expression analyses, Pearson’s correlation with a cut-off value of ≥0.8 was applied on all entities after filtering on entities that have been detected in at least one condition. Starch genes found to be highly expressed in tubers, which were *SuSy4*, *SBE3*, *GPT2.1*, were used as queries. If more than one valid probe was available, all probes were used as queries for the correlation and the resulting lists were reconciled using Venn-diagrams. Only entities correlating with all query features representing the same gene were considered.

Additional confirmative expression and co-expression analyses were conducted on a third independent set of samples which was derived from RNA-sequencing data available on the SpudDB website. The following samples were chosen for the analysis since they comprise untreated leaf and tuber tissues: BV_L [DM Leaves], S2 [RH Leaf], BV_P_S [DM Tubers (Whole, Sample 2)], S7 [RH Young Tuber], S8 [RH Mature Tuber] and S15 [RH Tuber Sprout]. For each starch gene FPKM values were extracted and further analyzed by calculating mean FPKM values for leaf and tuber tissue, respectively, and determining the ratio between the average values. Co-expression analysis was conducted in Excel by applying the function PEARSON on all genes using the same query genes as in the microarray analyses.

## Additional files

Additional file 1: Phylogenetic analysis of gene families involved in starch metabolism. Tree calculation was based on a global alignment with free end gaps, BLOSUM62 cost matrix and Jukes-Cantor genetic distance model. The tree was built by the Geneious 5.5.6 Tree Builder module employing a neighbour-joining method. a) alpha-amylases, b) beta-amylases, c) phosphoglucomutases, d) starch synthases, e) sucrose synthases, f) glucose-6-phosphate-phosphate translocators, g) starch branching enzymes, h) ADP-glucose pyrophosphorylases, i) isoamylases. The scale bar at the bottom represents the average substitutions per amino acid site. (PDF 110 kb)
Additional file 2: Valid microarray identifiers for starch genes in the POCI 4x44k and 8x60k platforms. (XLS 46 kb)
Additional file 3: Description of samples analyzed in this study. (XLS 36 kb)
Additional file 4: Heat Map representing fold-changes in gene expression levels of starch genes in leaf vs. tuber samples. (PDF 114 kb)
Additional file 5: Entities similar to SuSy4, GPT2.1 and SBE3 in a) POCI experiments, b) 8x60k experiments and c) RNA-Seq data. d) shows the genes commonly co-expressed in all platforms. (XLS 1177 kb)
Additional file 6: Primers used in this study. (XLS 38 kb)
Additional file 7: Normalized data all entities POCI 4x44k. (TXT 6594 kb)
Additional file 8: Normalized data all entities 8x60k. (TXT 11290 kb)

## Abbreviations

FPKM: Fragments Per Kilobase Of Exon Per Million Fragments Mapped
iTAG: International tomato annotation group
PGSC: Potato genome sequencing consortium
