# Supplementary figures and images for: Genome-wide analysis of starch metabolism genes in potato (Solanum tuberosum L.)
Source: BMC Genomics. 2017 Jan 5;18:37. doi: 10.1186/s12864-016-3381-z (PMC5217216; doi:10.1186/s12864-016-3381-z)

a)

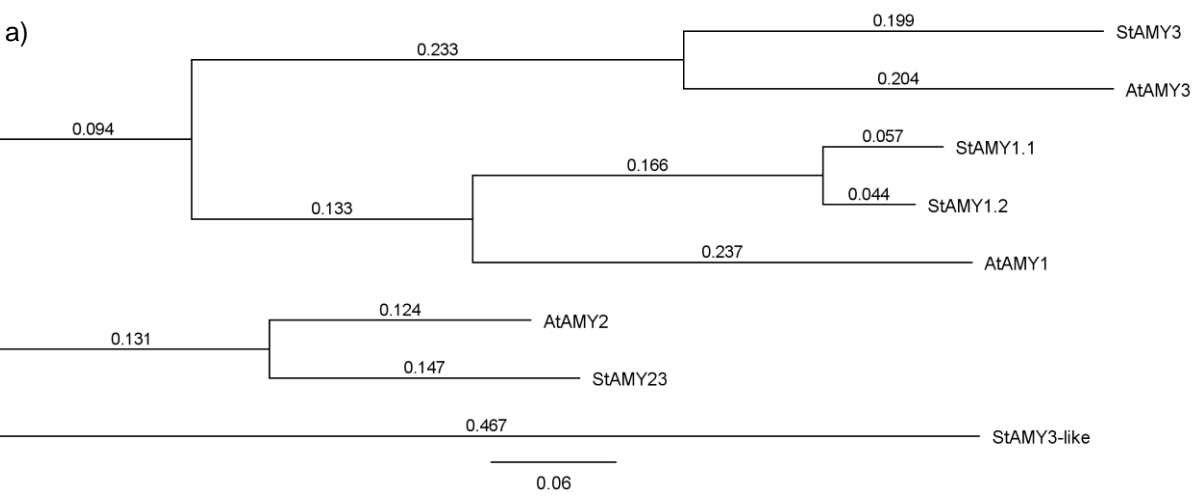

b)

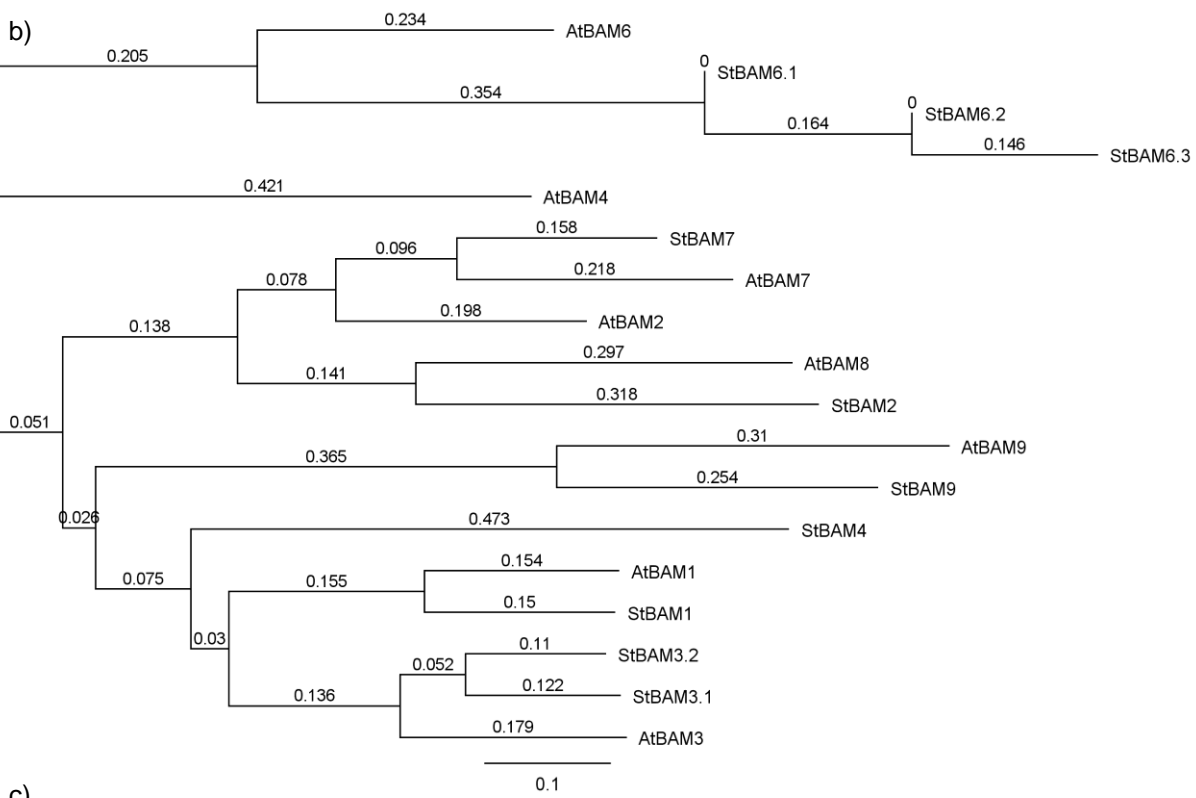

c)

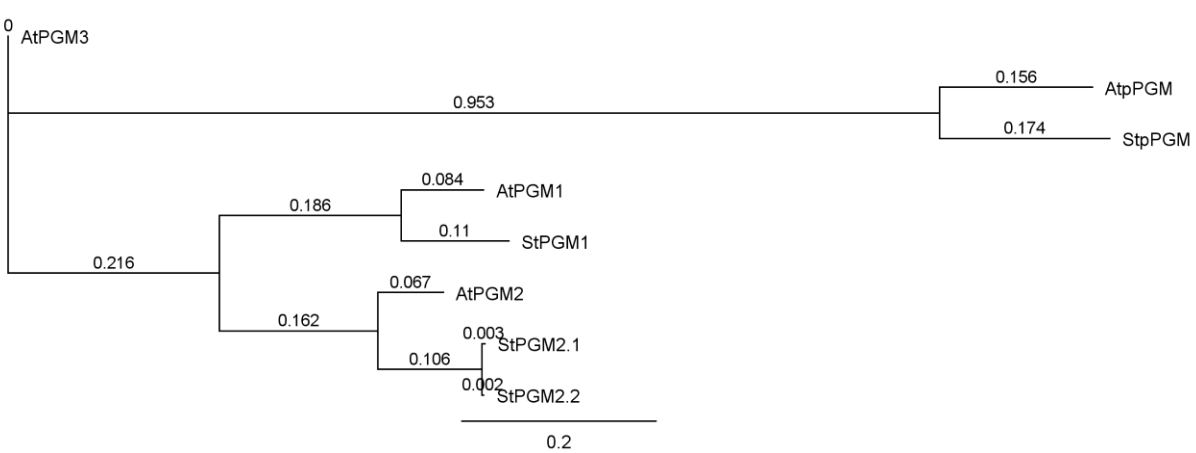

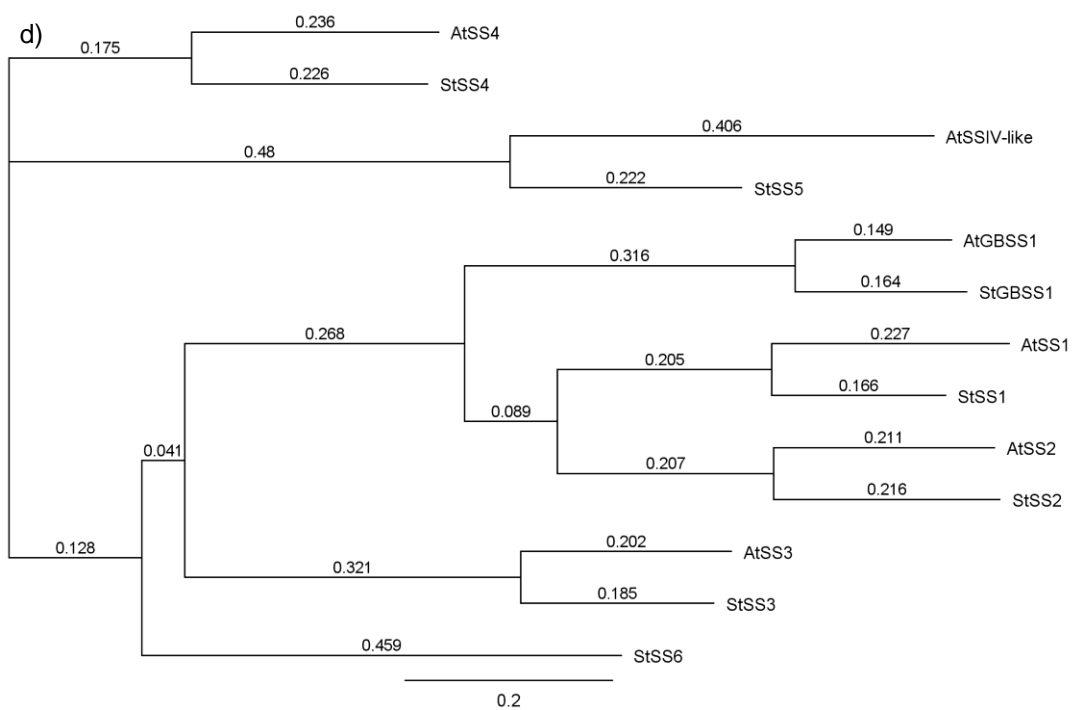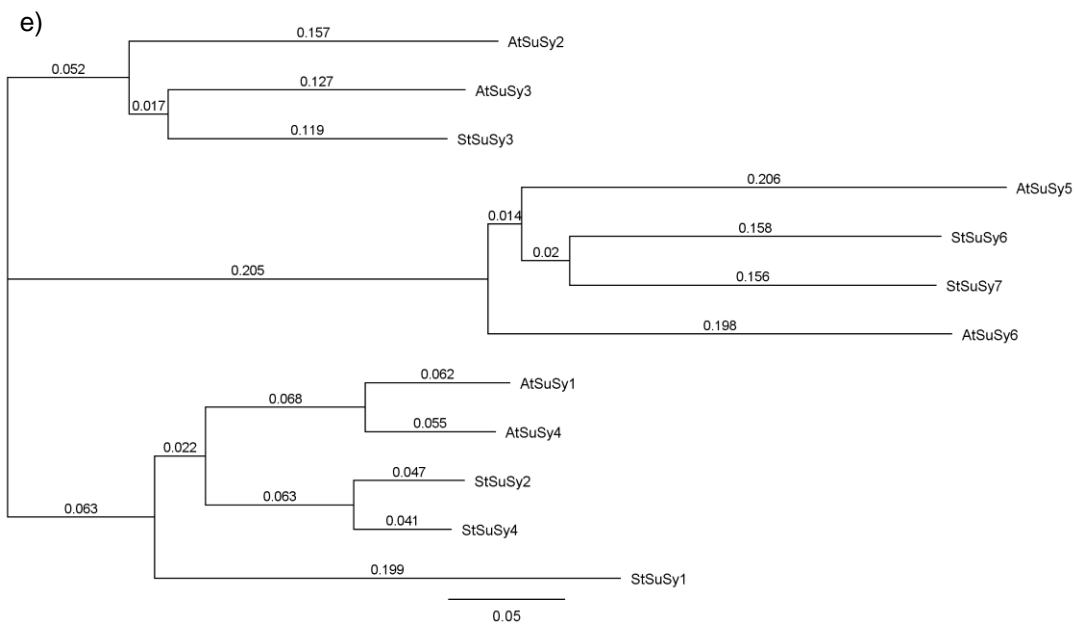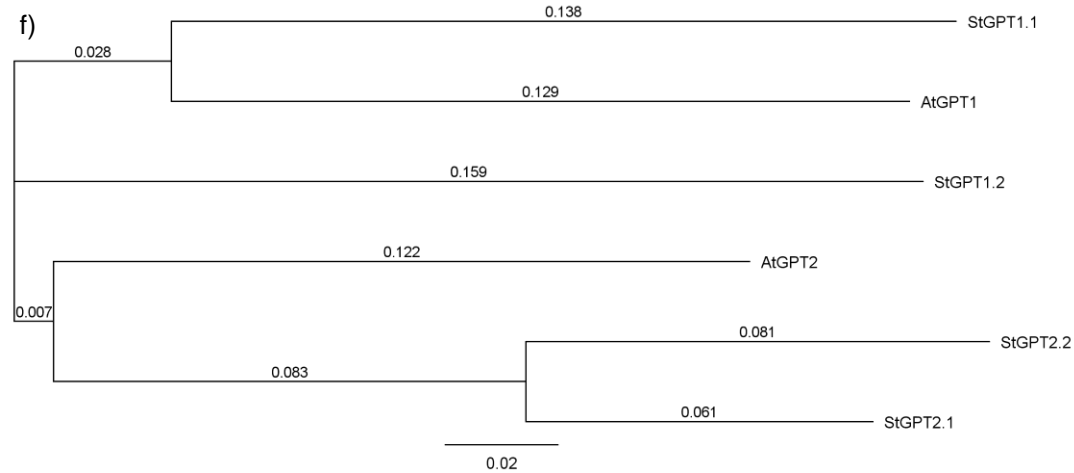

g)

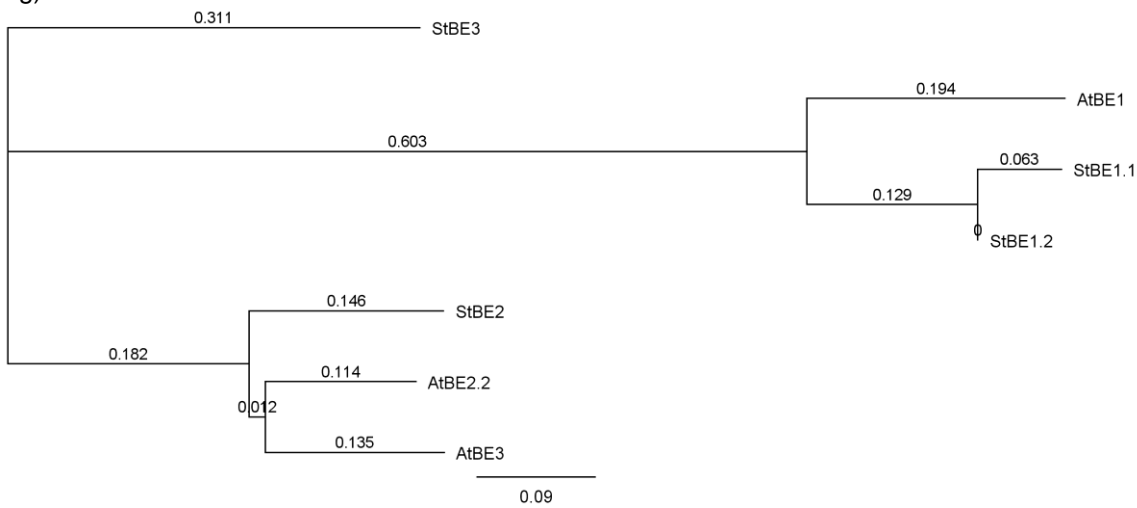

h)

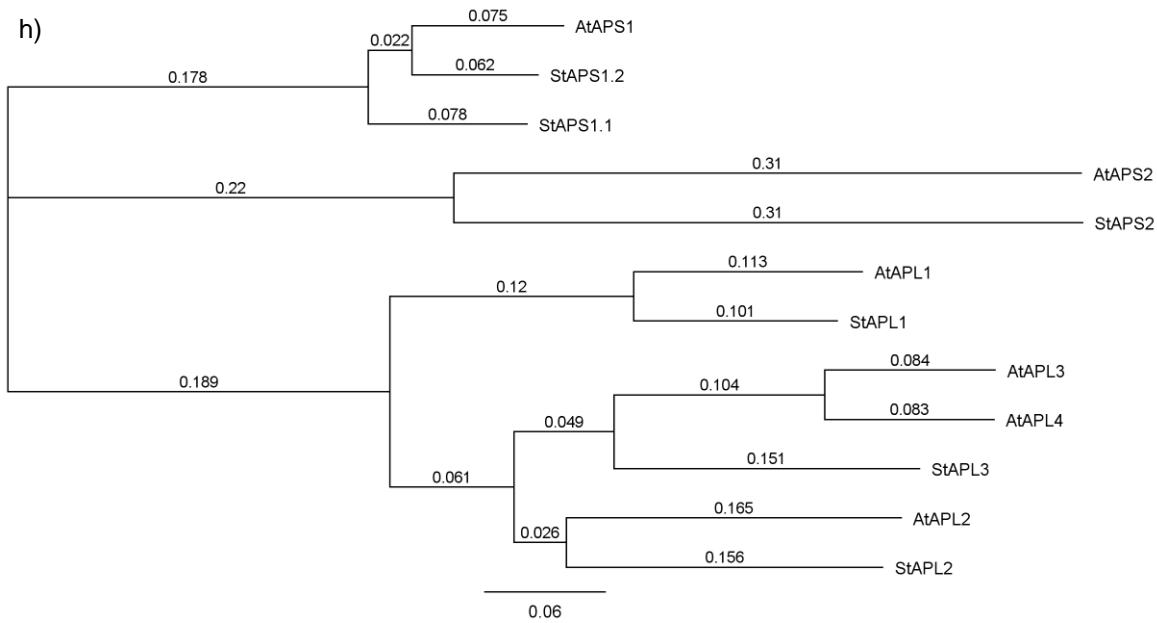

i)

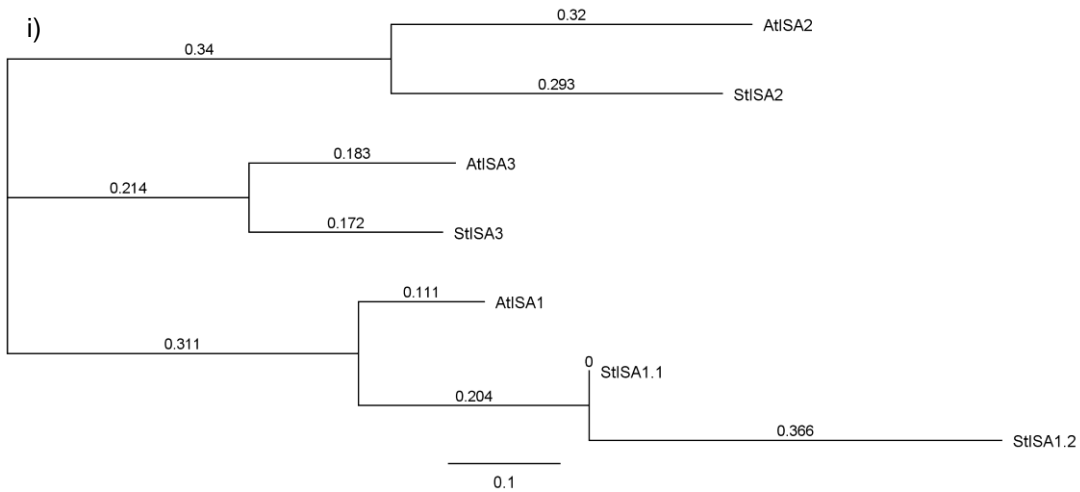

Supplement: Additional file 1: — Phylogenetic analysis of gene families involved in starch metabolism. Tree calculation was based on a global alignment with free end gaps, BLOSUM62 cost matrix and Jukes-Cantor genetic distance model. The tree was built by the Geneious 5.5.6 Tree Builder module employing a neighbour-joining method. a) alpha-amylases, b) beta-amylases, c) phosphoglucomutases, d) starch synthases, e) sucrose synthases, f) glucose-6-phosphate-phosphate translocators, g) starch branching enzymes, h) ADP-glucose pyrophosphorylases, i) isoamylases. The scale bar at the bottom represents the average substitutions per amino acid site. (PDF 110 kb) [file 12864_2016_3381_MOESM1_ESM.pdf]
