# Supplementary material for: Genome-wide analysis of starch metabolism genes in potato (Solanum tuberosum L.)
Source: BMC Genomics. 2017 Jan 5;18:37. doi: 10.1186/s12864-016-3381-z (PMC5217216; doi:10.1186/s12864-016-3381-z)

	FC leaf vs tuber		
Name	POCI	8x60k	FPKM
AMY1.1	14.48	12.01	6.89
AMY1.2	1.85	4.17	1.39
AMY23	-1.37	-3.33	-5.12
AMY3	2.86	6.31	5.02
APL1	34.45	22.80	321.89
APL2	-1.12	n.d.	-1.95
APL3	-7.17	-20.78	-16.73
APS1.1	-2.64	-7.05	-1.52
APS1.2	n.d.	-8.48	-4.06
APS2	n.d.	-1.66	n.d.
BAM1	2.40	2.18	1.94
BAM2	1.08	n.d.	-1.57
BAM3.1	4.73	79.36	7.00
BAM3.2	6.65	11.92	37.56
BAM4	0.97	0.00	-1.33
BAM6.1	n.d.	3.03	212.92
BAM6.2	n.d.	n.d.	63.77
BAM6.3	n.d.	n.d.	2.13
BAM7	n.d.	1.91	-1.19
BAM9	n.d.	-2.54	-5.96
DPE1	-6.07	-13.30	-7.42
DPE2	-1.87	n.d.	n.d.
GBSS1	-5.37	-16.07	-2.80
GLT1	0.00	0.24	-1.04
GPT1.1	-8.07	-29.05	-15.43
GPT2.1	-19.95	-1014.59	-405.99
GPT2.2	-1.25	17.53	-22.21
GWD	-6.65	-2.26	-1.00
PPase	1.16	1.26	2.84
PPase-like	2.55	1.05	3.52
ISA1.1	-5.82	n.d.	1.33
ISA1.2	n.d.	n.d.	1.87
ISA2	-2.43	-4.80	-1.38
ISA3	-1.43	-4.21	-1.44
LDE	-7.29	n.d.	n.d.
LSF1	-1.40	-2.72	1.07
LSF2	1.21	-1.86	-1.41
MEX1	-2.14	-1.02	2.97
NTT1	1.12	1.38	1.35
NTT2	-10.45	-18.53	-6.74
PGI	1.34	-1.33	1.36
PGI-like1	1.21	n.d.	n.d.
PGI-like2	n.d.	-1.29	1.97
PGM1	-5.45	n.d.	n.d.
PHO1a	-11.16	-12.33	-11.92
PHO1b	13.64	2.76	227.11
PHO2a	-3.28	n.d.	n.d.
PHO2b	0.59	1.49	-1.33
PWD	-0.69	-0.33	1.52
SBE1.1	n.d.	n.d.	1.92
SBE2	1.39	n.d.	n.d.
SBE3	-9.48	-30.56	-18.01
SEX4	-9.95	-103.78	-18.12
SEX4-like	n.d.	n.d.	1.02
SS1	1.45	2.06	8.49
SS2	-2.01	-4.61	-1.80
SS3	-2.26	-2.71	-1.22
SS4	1.93	1.58	1.72
SS5	-12.21	-96.00	-33.73
SS6	1.70	n.d.	-3.23
SuSy1	-5.63	-24.82	-23.59
SuSy2	-3.92	-2.09	-36.17
SuSy3	-5.96	-5.69	-9.53
SuSy4	-88.80	-662.28	-1024.66
SuSy6	-1.32	-4.55	-2.70
SuSy7	-1.42	-5.77	-2.98
TPT	n.d.	12.68	33.41
VGT3-like	n.d.	3.33	n.d.

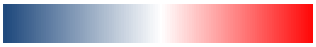

Supplement: Additional file 4: — Heat Map representing fold-changes in gene expression levels of starch genes in leaf vs. tuber samples. (PDF 114 kb) [file 12864_2016_3381_MOESM4_ESM.pdf]
